# The neuronal transcription factor MEIS2 is a calpain-2 protease target

**DOI:** 10.1242/jcs.261482

**Published:** 2024-02-28

**Authors:** Tanja Müller, Marina Reichlmeir, Ann-Christin Hau, Ilka Wittig, Dorothea Schulte

**Affiliations:** ^1^ Goethe University, Faculty of Medicine, University Hospital Frankfurt, Institute of Neurology (Edinger Institute), 60528 Frankfurt, Germany; ^2^ Goethe University, University Hospital Frankfurt, Dr. Senckenberg Institute of Neurooncology and Institute of Neurology (Edinger Institute), Frankfurt Cancer Institute (FCI), University Cancer Center Frankfurt (UCT), MSNZ Junior Group Translational Neurooncology, 60528 Frankfurt, Germany; ^3^ Department of Cancer Research (DoCR), Luxembourg Institute of Health (LIH), Luxembourg Centre of Neuropathology (LCNP), 1445 Luxembourg, Luxembourg; ^4^ Goethe University, Faculty of Medicine, Institute for Cardiovascular Physiology, Functional Proteomics, 60590, Frankfurt, Germany

**Keywords:** Calpain, MEIS homeodomain protein, Subventricular zone, Stem cell, Adult neurogenesis, Transcription factor

## Abstract

Tight control over transcription factor activity is necessary for a sensible balance between cellular proliferation and differentiation in the embryo and during tissue homeostasis by adult stem cells, but mechanistic details have remained incomplete. The homeodomain transcription factor MEIS2 is an important regulator of neurogenesis in the ventricular–subventricular zone (V-SVZ) adult stem cell niche in mice. We here identify MEIS2 as direct target of the intracellular protease calpain-2 (composed of the catalytic subunit CAPN2 and the regulatory subunit CAPNS1). Phosphorylation at conserved serine and/or threonine residues, or dimerization with PBX1, reduced the sensitivity of MEIS2 towards cleavage by calpain-2. In the adult V-SVZ, calpain-2 activity is high in stem and progenitor cells, but rapidly declines during neuronal differentiation, which is accompanied by increased stability of MEIS2 full-length protein. In accordance with this, blocking calpain-2 activity in stem and progenitor cells, or overexpression of a cleavage-insensitive form of MEIS2, increased the production of neurons, whereas overexpression of a catalytically active CAPN2 reduced it. Collectively, our results support a key role for calpain-2 in controlling the output of adult V-SVZ neural stem and progenitor cells through cleavage of the neuronal fate determinant MEIS2.

## Teaser

**Highlighted Article:** The intracellular protease calpain-2 modulates neurogenesis of adult neural stem and progenitor cells of the mouse forebrain through cleavage of the neuronal transcription factor MEIS2.

## INTRODUCTION

Eukaryotic cells exhibit a remarkable number of genetic responses to their environment. This is largely the result of transcription factors (TFs) and their ability to control gene expression in a spatiotemporal manner. Tightly balanced TF networks are therefore a prerequisite for all physiological processes in the healthy organism, whereas their dysregulation has been linked to human diseases, including cancer (Furney et al., 2006). Genome-wide approaches such as ChIP-sequencing, ATAC-sequencing and RNA-sequencing, both at the single-cell and on the population level, have greatly advanced our knowledge about how transcription factors act. Much less, however, is known about how the activity of TFs itself is regulated. RNA stability, the rate of protein synthesis or degradation, controlled import or export from the cell nucleus, and post-translational modifications (PTMs) are all important post-transcriptional and post-translational levers by which the activity of TFs can be controlled and quickly adapted to a changing extracellular environment.

Myeloid ecotropic viral integration site 2 (MEIS2) encodes for a TF of the MEINOX family, a separate group within the atypical ‘three amino acid loop extension’ (TALE) superclass of homeodomain (HD)-containing proteins (Mukherjee and Bürglin, 2007; Schulte and Geerts, 2019). Phenotypes associated with the deletion or overexpression of the *Meis2* gene in animal models have revealed that *Meis2* participates in craniofacial and cardiac development (Machon et al., 2015; Paige et al., 2012; Wang et al., 2020a), outgrowth and patterning of the limbs (Delgado et al., 2020, 2021), and patterning of the axial skeleton (López-Delgado et al., 2020). *Meis2* also contributes to virtually all aspects of central nervous system development, including neural tube patterning, neural progenitor cell proliferation, cell-fate acquisition, neuronal maturation, neurite outgrowth and synaptogenesis (Agoston and Schulte, 2009; Conte et al., 2010; Dupacova et al., 2021; Heine et al., 2008), and is required for the survival and function of diverse populations of neurons (Frazer et al., 2017; Jakovcevski et al., 2015). *MEIS2* has further been identified as susceptibility gene for obsessive-compulsive behavior in humans, and somatic mutations creating *de novo* MEIS-binding motifs have been discovered in putative enhancer elements in brains from individuals diagnosed with autism spectrum disorder (Nestadt et al., 2012; Roussel et al., 2022). Consistent with these phenotypes, heterozygous *MEIS2* missense mutations or 15q14 microdeletion involving the *MEIS2* gene locus are characterized by a triad of cleft palate, atrial or ventricular septal heart defects, and intellectual disability, known as *MEIS2* syndrome (Verheije et al., 2019).

MEIS2 is also an important regulator of adult neurogenesis in the ventricular-subventricular zone (V-SVZ) of mice (Agoston et al., 2014). Neurogenesis in the V-SVZ progresses through a series of cellular states, from quiescent and activated neural stem cells (NSCs) over an intermediate, highly reproductive stage of transient amplifying progenitor cells (TAPs) to neuroblasts. Most neuroblasts migrate into the olfactory bulb (OB) where they mature to distinct types of GABAergic interneurons, integrate into existing neuronal networks and contribute to the continuous adjustment of the olfactory circuitry (Bragado Alonso et al., 2019). The transitions between each of these states are subject to tight epigenetic and transcription factor-driven control (Obernier and Alvarez-Buylla, 2019). MEIS2 is one of the transcription factors that initiate neuronal differentiation programs in this system (Hau et al., 2017). Close regulation over its expression, subcellular localization and activity is therefore crucial for adult V-SVZ neurogenesis.

Proteolytic cleavage is the irreversible enzymatic hydrolysis of peptide bonds within substrate proteins and an important PTM to regulate protein function or turnover. Calpains are a family of 15 calcium (Ca^2+^)-dependent cysteine proteases, which are widely expressed in eukaryotic cells with both conventional and non-conventional subtypes (Sorimachi et al., 2011). Calpain-2 (also known as m-calpain), belonging to the family of conventional calpains, is a heterodimer consisting of the large subunit CAPN2 and the small regulatory subunit CAPN4 (also known as CAPNS1). Conventional calpains cleave various structural proteins and components of intracellular signaling pathways (Baudry and Bi, 2016; Perrin and Huttenlocher, 2002). Their enzymatic activity is modulated by Ca^2+^, dimerization with the small subunit CAPN4, the endogenous inhibitor calpastatin (CAST) and PTMs. Calpains proteolyze their substrate proteins in a limited manner, a process known as ‘proteolytic processing’. This processing differs fundamentally from the immediate and complete degradation that is carried out by the proteasome, lysosome, or during apoptosis or autophagy (Ono and Sorimachi, 2012). Calpain cleavage products can become further degraded or might be stably retained as neo-polypeptides and take on physiological functions that differ from those of the unprocessed protein (Ono and Sorimachi, 2012). As an example for the former, ten-eleven translocation (TET) enzymes, key guardians of DNA methylation patterns, are cleaved by calpain-1 or calpain-2, initiating the controlled turnover of TET proteins in mouse embryonic stem cells (mESCs) and during mESC differentiation, respectively (Wang and Zhang, 2014). An example for a calpain substrate with a *de novo* function is the oncogenic transcription factor MYC (Shapovalov et al., 2022). MYC cleavage by calpains produces a transcriptionally inactive fragment (MYC-Nick), which is retained in the cytoplasm and contributes to various physiological and pathophysiological processes (Conacci-Sorrell et al., 2010). Calpains have been linked to the differentiation of several cell types including myoblasts (Dourdin et al., 1999), pre-adipocytes (Yajima and Kawashima, 2002) and pre-osteoblasts (Murray et al., 1997), and have been linked to cancer (Shapovalov et al., 2022; Storr et al., 2011). Targeted deletion of CAPN4 impairs adult neurogenesis in the hippocampus, neuronal survival and synaptic function (Amini et al., 2013; Baudry and Bi, 2016). Calpain-2, in turn, has a role in neurodegeneration, and calpain-2 inhibition has been identified as a promising treatment for mitigating the effects of concussion-induced neuropathy (Wang et al., 2020b). The contribution of calpain-2 to adult neurogenesis, however, is largely unexplored, and calpain-2 substrates have remained enigmatic in most cases.

Here, we found that calpain-2 cleaves MEIS2 and impacts neurogenic differentiation in a process that is regulated by phosphorylation of the MEIS2 protein and prevented by MEIS2–PBX1 heterodimerization. Our results thus link a Ca^2+^-dependent proteolytic system in adult neural stem cells to the turnover of a transcriptional regulator of neurogenesis and provide an explanation for how Ca^2+^ dynamics might directly impact on the neurogenic output of resident stem and progenitor cells in the adult V-SVZ stem cell niche.

## RESULTS

### Calpain-2 protease activity is high in primary adult neurospheres but decreases with differentiation

**Fig. 1.**
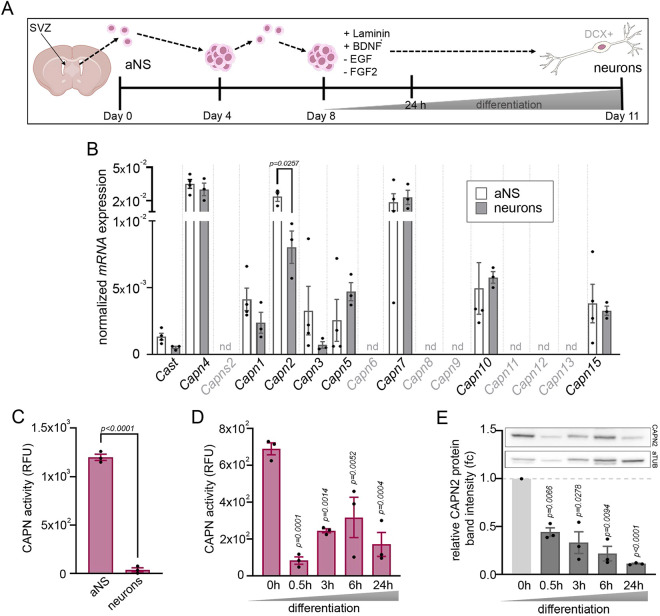
**Calpain-2 protease activity is high in primary aNS and downregulated upon differentiation.** (A) Schematic representation of the neurosphere assay. (B) Normalized transcript levels of calpain proteases in aNS and neurons. *n*=3. nd, no transcript detected. *P*-values are indicated [one-way ANOVA multiple comparison analysis (MCA; Tukey's post test)]. (C) Absolute calpain activity in whole-cell extracts of aNS cells compared to *in vitro* differentiated neurons expressed as relative fluorescence units (RFU). aNS *n*=4; neurons *n*=3. *P*-value is indicated (unpaired two-tailed *t*-test). (D) Calpain activity measurements (RFU) during *in vitro* differentiation of aNS. *n*=3. *P*-values are indicated (one-way ANOVA MCA, each timepoint compared to 0 h). (E) Representative immunoblots of CAPN2 protein during *in vitro* differentiation and densitometric analysis of *n*=3. Differences expressed as fold change (fc) relative to the 0 h value (set at 1). *P*-values are indicated (one sample *t*-test). All data are represented as mean±s.e.m.; *n*=biological replicates.

Previous studies have observed particularly active Ca^2+^ transients in the adult V-SVZ stem cell niche and reported that these Ca^2+^ dynamics influence stem cell behavior under physiological and pathophysiological conditions (Beckervordersandforth et al., 2010; Kraft et al., 2017; Lacar et al., 2011). One way by which fluctuating Ca^2+^ levels might affect the neurogenic output of stem cells would be through an influence of calpain family proteases on the activity of neurogenic TFs. Here, we tested this hypothesis with the help of an *in vitro* system of acutely dissected, primary stem and progenitor cells. The so called *in vitro* neurosphere assay is an effective technique to study molecular mechanisms related to neural stem cell maintenance and neurogenesis (Soares et al., 2021). NSCs and TAPs isolated from the mouse V-SVZ can be expanded in the presence of epidermal growth factor (EGF) and fibroblast growth factor 2 (FGF2) as free-floating three-dimensional aggregates, termed adult neurospheres (aNS), and will differentiate into neurons, astrocytes and oligodendrocytes when plated on laminin in medium lacking EGF and FGF2 (Fig. 1A). By retroviral expression of the neurogenic transcription factor *Pax6* and supplementing the medium with brain-derived neurotrophic factor (BDNF), cellular differentiation can be substantially shifted towards neurogenesis (Hack et al., 2004). We quantified calpain transcript, protein and activity in aNS and young neurons differentiated from these. Of the 15 known Capn family genes, *Capn2*, *Capn7* and the regulatory subunit *Capn4* were strongly expressed in aNS. *Capn1*, *Capn5*, *Capn10* and *Capn15* exhibited medium to low expression levels, whereas the remaining family members were not detected (Fig. 1B). The transcript levels of all Capn family genes that are expressed in aNS remained stable when the cells were subjected to PAX6-induced neuronal differentiation *in vitro*, except *Capn2* whose expression was markedly lower in neurons compared to aNS (Fig. 1B). The endogenous calpain inhibitor *Cast* was barely detectable, neither in aNS nor neurons. This is noteworthy, as a genetic deletion model of *Cast* had previously served to investigate the contribution of calpains to adult V-SVZ neurogenesis (Machado et al., 2015). However, given its low expression, we consider it unlikely that *Cast* is relevant for any function that the calpain proteolytic system might have in primary aNS and during their neuronal differentiation. We therefore focused on *Capn2*, the only calpain that exhibits significantly different expression in aNS compared to neurons. Calpain activity measurements with the fluorescent calpain sensor ACM substrate revealed that calpain activity in aNS extracts was several orders of magnitude higher than in extracts of *in vitro* differentiated neurons (Fig. 1C). Calpain activity decreased significantly within the first 30 min after induction of neuronal differentiation (Fig. 1D). Considering that aNS predominantly express *Capn2* (Fig. 1B), the drop in calpain activity likely originates from calpain-2, even though the calpain substrate used in this activity assay can be cleaved by calpain-1 and calpain-2. Consistent with calpain-2 playing a major role in adult V-SVZ neurogenesis, CAPN2 protein levels decreased rapidly when *in vitro* differentiation was induced and remained at low levels throughout a 24 h observation period (Fig. 1E). In addition, treating aNS with pharmacological agents that induce cellular differentiation when administered to the adult V-SVZ *in vivo* or to aNS *in vitro*, such as inhibitors of EGF receptor (EGFR) (Craig et al., 1996; Doetsch et al., 2002), protein kinase C (PKC) (García-Bernal et al., 2018) or MEK1 and MEK2 (MEK1/2, also known as MAP2K1 and MAP2K2) (Lee et al., 2019), also resulted in a significant reduction of calpain activity (Fig. S1). High calpain activity is thus a hallmark of undifferentiated cells in the neurosphere assay.

### Inhibition of calpain-2 enhances and activation of calpain-2 reduces *in vitro* neuronal differentiation of primary aNS

**Fig. 2.**
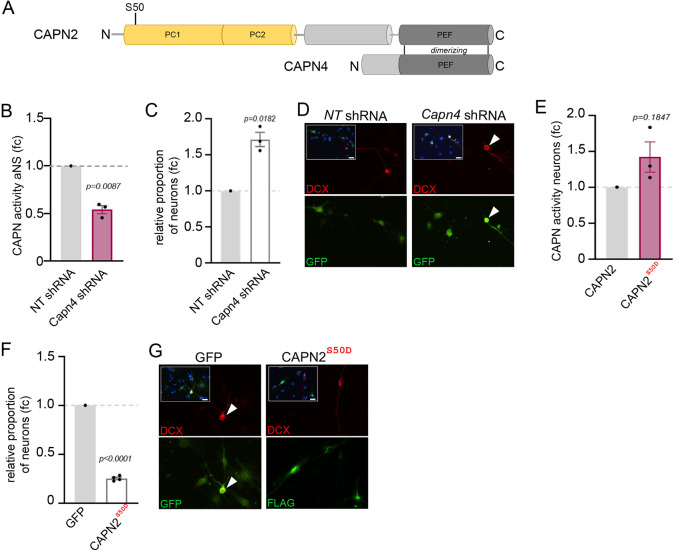
**Inhibition of calpain-2 enhances whereas its activation reduces *in vitro* neuronal differentiation of aNS.** (A) Schema of CAPN2 and CAPN4 domains. PC, protease domain; PEF, penta-EF-hand domain; FLAG, FLAG tag. Phosphosite S50 is indicated, the constitutively active mutation is S50D. (B) Calpain activity (RFU) in cell extracts of aNS transduced with *Capn4* shRNA or non-targeting (NT) control shRNA. *n*=3. (C) Relative proportion of DCX+ neurons following transduction of *Capn4* shRNAs expressed as fold change (fc) relative to NT shRNA control. *n*=3. (D) Representative images of DCX immunostaining (red) in cell transduced with *Capn4* or NT shRNA visualized by GFP (green). White arrowheads mark double-positive cells; inserts, merged DCX, GFP and DAPI channels. Scale bars: 10 μm. (E) Calpain activity (RFU) in cell extracts of aNS transduced with CAPN2 or CAPN2^S50D^ followed by neuronally directed differentiation; *n*=3. (F) Relative proportion of DCX+ neurons following CAPN2^S50D^ transduction expressed as fc relative to transduction with empty vector (GFP); *n*=4. (G) Representative images of colocalization of DCX with GFP in cells transduced with the empty vector or co-localization of DCX with FLAG (green) in cells transduced with CAPN2^S50D^ fused to a FLAG-epitope. White arrowheads mark double-positive cells; inserts: merged DCX, FLAG and DAPI channels. Data are represented as mean±s.e.m. *P*-value is indicated (B, C, E and F; parametric one sample *t*-test). *n*=biological replicates. Scale bars: 10 μm.

Calpains carry out many cellular functions, performing, among other functions, important roles in the initiation, regulation and execution of cell death (Harwood et al., 2005). Cell death is less of a problem in *in vitro* studies, because these can be performed quickly and monitored during the experiment, but it can profoundly complicate the interpretation of *in vivo* studies, such as in transgenic mouse models or intracranial application of pharmacological agents or viruses. To circumvent these potential issues, we turned to *in vitro* assays for subsequent functional studies. Formation of a proteolytic active calpain-2 holoenzyme requires dimerization of CAPN2 with the small regulatory subunit CAPN4 and alignment of amino acids within the penta-EF-hand domains (Moldoveanu et al., 2002) (Fig. 2A). shRNA-mediated knockdown of *Capn4*, but not transduction with non-targeting (NT) shRNAs, significantly lowered the calpain activity of aNS extracts (Fig. 2B). Interestingly, aNS depleted for *Capn4* generated nearly twice as many neurons, detected by staining for the neuron-specific marker doublecortin (DCX+), upon *in vitro* differentiation than cells transfected with the NT control shRNAs (Fig. 2C,D). We also took advantage of the fact that the enzymatic activity of calpain-2 is subject to PTMs on the CAPN2 subunit. Among these PTMs, phosphorylation on serine 50 (S50) increases calpain-2 activity independently of the prevailing intracellular Ca^2+^ concentration (Glading et al., 2004). Replacing S50 with the phospho-mimetic glutamic acid in CAPN2 (CAPN2^S50D^) thus generates a calpain protease that is constitutively active and largely insensitive towards small to moderate fluctuations in Ca^2+^ concentration (Glading et al., 2004). Overexpression of this constitutive active calpain in aNS prior to differentiation not only led to persistently high calpain cleavage activity (Fig. 2E) and thereby bypassed the drop in endogenous calpain activity that normally accompanies cellular differentiation (Fig. 1D), but also significantly reduced the number of DCX+ neurons produced from transduced cells (Fig. 2F,G). Experimentally reducing or increasing calpain-2 activity in aNS thus markedly affected their ability to generate neuronal progeny.

### Differences in protein stability of MEIS2 transcription factor in neural progenitor cells and young neurons

**Fig. 3.**
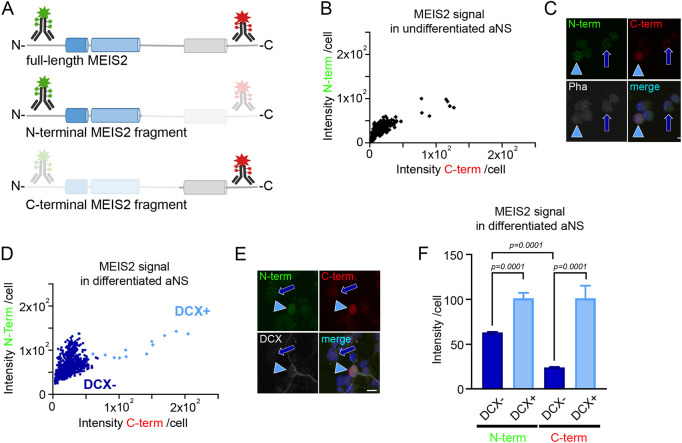
**MEIS2 in aNS and differentiating neurons.** (A) Schematic overview of the antibody staining applied in this figure. Two antibodies, directed against the N- and C-terminus of MEIS2 have been used. Staining intensities of the N-terminal antibody and C-terminal antibody within single cells were analyzed and compared. (B) Scatter blot of single aNS fluorescence intensity values of cells immunostained with C- and N-terminal MEIS2-specific antibodies. Values are expressed as mean intensity values per cell nucleus; data points represent all cell nuclei assessed, from 230 cells. The few cells appearing shifted towards higher intensity values likely correspond to cells undergoing spontaneous differentiation. (C) Representative confocal images of analyzed cells counterstained with cytoplasmic marker phalloidin (Pha); dark blue arrow, undifferentiated cells weakly stained with N- and C-terminal MEIS2 antibody; light blue arrowhead, cell undergoing spontaneous differentiation displaying increased N- and C-terminal MEIS2 staining intensity. (D) Scatter blot of single cell fluorescence intensity values of cells stained with antibodies specific for C- or N-terminal MEIS2 or DCX, respectively, after 24 h of differentiation. Values are expressed as mean intensity values per cell nucleus; data points represent all cell nuclei assessed, from 980 cells. (E) Representative confocal images; dark blue arrow, immunoreactivity in a DCX− cell weakly stained with N- and C-terminal MEIS2 antibody; light blue arrowhead, immunoreactivity in a DCX+ cell showing increased N- and C-terminal MEIS2 staining intensity. (F) Mean intensity values per cell nucleus after automated quantification for N- and C-terminal MEIS2 antibody staining of the cells plotted in D, mean±s.e.m., *n*=F DCX+, 17 cells; DCX−, 963 cells; the cells were analyzed from eight samples in one experiment. *P*-values are indicated (Mann–Whitney *U*-test). Scale bars: 10 μm.

Many neuronal genes already exhibit remarkably high levels of transcript expression in V-SVZ stem cells, but the dynamics of the corresponding proteins are not well understood (Beckervordersandforth et al., 2010). A good example for such a neuronal gene is *Meis2*. *Meis2* transcripts can be readily detected in *GFAP-CD133*-expressing neural stem cells in the V-SVZ, but MEIS2 immunoreactivity only emerges when cells progress towards neurogenesis (Lepko et al., 2019). We therefore set out to assess MEIS2 protein in aNS in a semi-quantitative manner by a combination of immunofluorescence staining and automated quantification of fluorescence intensities. For increased precision of our measurements, two antibodies, directed against the N- and C-terminus of MEIS2 respectively, were applied (Fig. 3A). Free-floating aNS, and, hence, cells grown under culture conditions that counteract cellular differentiation, were only weakly stained by both MEIS2 antibodies (Fig. 3B,C, arrows). The few cells exhibiting more intense MEIS2 staining might correspond to the small fraction of cells that undergo spontaneous neuronal differentiation even in the presence of EGF and FGF2 (Azari and Reynolds, 2016) (Fig. 3B,C, arrowheads). However, at 24 h after induction of cellular differentiation by EGF and FGF2 withdrawal, both N- and C-terminal antibodies strongly stained the nuclei of DCX-positive (DCX+ cells; light blue or arrowheads) but not the nuclei of DCX-negative cells (DCX−; dark blue or arrows) , as shown in Fig. 3D,E. DCX marker-negative cells at this early time of differentiation either correspond to remaining neural progenitor cells, cells adopting a non-neuronal fate or neuronally committed cells prior to DCX expression. Of note, when the signal intensity values obtained with the two antibodies were compared, two remarkable differences between DCX− and DCX+ cells became apparent (Fig. 3F). First, unbiased software-based analyses revealed that signal intensity values measured in DCX− cells for both N- and C-terminal MEIS2 were generally lower than values for DCX+ cells, indicating that neuronal differentiation is accompanied by stabilization and/or upregulation of MEIS2. Second, the values obtained with the N- and C-terminally directed antibodies were nearly identical in DCX+ cells, as would be expected for two antibodies that bind to the same polypeptide, but differed markedly in DCX− cells. This observation can be best explained by the assumption that the MEIS2 N- and C-termini exist separately from each other in DCX− cells and consequently are recognized independently by the two antibodies. C-terminal cleavage products thereby appear to be less well retained in the cells compared to N-terminal cleavage fragments, as evident by the fact that the intensity values of the DCX− cell cohort (dark blue) are visibly shifted towards the *y*-axis (Fig. 3D). In DCX+ cells (light blue), by contrast, intact MEIS2 protein appears to predominate, which is detected by both antibodies with comparable efficiencies. We concluded from these measurements that MEIS2 protein stability negatively correlates with calpain protease activity.

### MEIS2 is a direct target of calpain-2 proteolytic cleavage

**Fig. 4.**
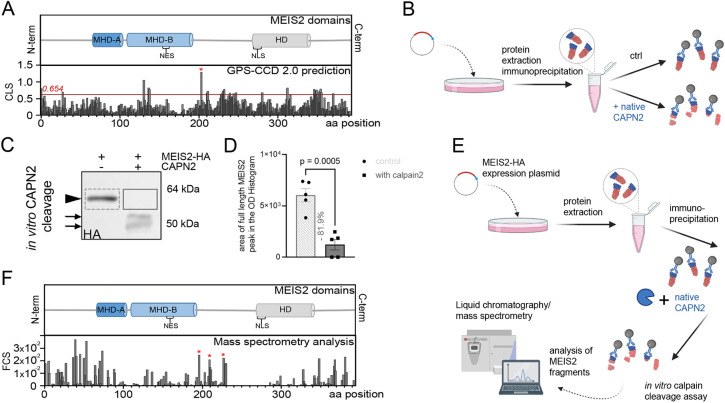
**MEIS2 is a direct target of calpain-2.** (A) Upper panel, MEIS2 protein domain structure. Conserved domains, MEINOX homology domain A (MHD-A), MEINOX homology domain B (MHD-B), nuclear export signal (NES), the homeodomain (HD) and nuclear localization signal (NLS) are marked. Lower panel, same scale graphical presentation of predicted calpain cleavage sites within MEIS2. Predictions were made using the GPS-CCD 2.0 tool. The *y*-axis represents the cleavage likelihood score (CLS) at respective amino acid positions. The red line at prediction score 0.654 indicates the maximum default cut-off value above which cleavage is assumed likely. (B) Schematic representation of the comparative calpain cleavage assays as assessed by immunoblotting. (C) Representative western blot of MEIS2–HA fragmentation following incubation with calpain-2 (solid gray box) or control without calpain-2 (dashed gray box). Immunoblot for HA epitope. Arrowhead, respective full-length proteins detected by HA-specific antibodies. Arrows, fragments detected upon incubation with calpain-2. Sizes of marker protein bands are indicated on the right. (D) Quantification of full-length MEIS2 signal on western blots following incubation with calpain-2 or control without calpain-2. For quantification, the area of full-length MEIS2 peak on optical density (OD) histogram of the respective lane was analyzed (mean±s.e.m.; *n*=5 biological replicates). Data are represented as mean±s.e.m. *P*-value is indicated (Wilcoxon one sample *t*-test). (E) Schematic representation of the experimental procedure for mass spectrometric identification of MEIS2 cleavage fragments. HA-tagged MEIS2 proteins were incubated with proteolytically active calpain-2, cleavage products were directly assessed by mass spectrometry. Blue, HA tag; red, MEIS2 portion of the fusion protein. (F) Upper panel: graphical representation of the MEIS2 protein domain structure. Lower panel: same scale graphical presentation of MEIS2 cleavage sites detected by mass spectrometry analysis (one experiment). The *y*-axis represents the frequency of cleavage sites (FCS) at the respective positions. Red asterisks mark sites referred to in the text. *x*-axis, MEIS2 amino acid positions.

This correlation motivated us to investigate whether MEIS2 might be a direct target of calpain-2 protease in aNS neurogenesis. Calpains lack a clear cleavage sequence specificity but recognize the overall conformation of their targets instead. It is therefore not possible to directly infer from the amino acid sequence of a protein at which position(s) cleavages by calpains could occur. However, based on the analysis of known calpain cleavage sites, the amino acid preferences around the cleavage bond in the substrate can be deduced and modeling was developed based on this (Liu et al., 2011). To get first clues about whether MEIS2 might be sensitive to proteolysis by calpain-2, we performed computer-based analysis of the MEIS2 amino acid sequence with the open-source software GPS-CCD (group-based prediction system – calpain cleavage detector; Liu et al., 2011). At total of 23 sites with a calpain cleavage likelihood score (CLS) above the maximum default cut-off value of 0.654 were identified. Of these sites, cleavage after amino acids at position 204, located within a largely unstructured region of the protein, had a particularly high score, indicating that this part of the MEIS2 amino acid chain might be particularly susceptible to calpain cleavage (Fig. 4A, CLS, red asterisk). To directly test whether MEIS2 is sensitive to calpain-2, we produced C-terminally HA-tagged MEIS2 protein by transfection of expression plasmids carrying *Meis2-HA* in HEK 293T cells and isolated MEIS2–HA by immunoprecipitation with HA-specific antibodies followed by elution of native proteins with the help of HA peptides. Protein isolates were divided into two equal aliquots, of which one was treated with proteolytically active porcine calpain-2 and the other one by the diluent but lacking calpain-2 (ctrl.) (Fig. 4B). From there on, both samples were treated identically and assessed for the cleavage of MEIS2 by immunoblotting with antibodies directed against the HA tag. Calpain-2 treatment cleaved the full-length MEIS2–HA protein (arrowhead) to several smaller fragments (Fig. 4C, arrows). Because only cleavage products that retain the HA tag can be visualized in this assay, we quantified the efficiency of calpain-2 cleavage on the immunoblot by comparing the optical density of the MEIS2–HA full-length protein band of samples with and without calpain incubation. Calpain-2 treatment drastically reduced the band intensity of full-length MEIS2 by 81.9% of the band intensity in the control sample (Fig. 4D). GST, a protein that does not contain predicted calpain cleavage sites as determined by GPS-CDD was not fragmented by calpain-2 under identical experimental conditions (Fig. S2A,B).

For further experimental validation of MEIS2 cleavage sites, cleavage products from purified MEIS2 subjected to calpain-2 treatment were analyzed by mass spectrometry. We devised a protocol by which fragmentation of the protein isolate was achieved by incubation with proteolytically active native porcine calpain-2 protease instead of the standard trypsin digestion step during sample preparation (Fig. 4E). By this approach, only protein fragments that are generated by calpain-processing are analyzed. Mapping the amino acid sequences of these cleavage products back onto the MEIS2 protein sequence allowed us to draw conclusions about the approximate position where the calpain cleavage must have occurred in the substrate protein, expressed as frequency of cleavage sites (FCS; Fig. 4F). Three regions of the MEIS2 protein exhibited particularly high FCS scores – the largely unstructured protein domains of the MEIS2 N- and C-terminus, as well as positions 196, 209 and 227, which are in a protein domain overlapping with the computer-predicted calpain-cleavage site (red asterisks in Fig. 4A). Cleavage between positions 196 to 227 separates two large α-helical domains within the MEIS2 protein, the bipartite MEIS homology domains MHD-A and MHD-B, through which MEIS2 interacts with its heterodimerization partner PBX1 (Bruckmann et al., 2020; Ryoo and Mann, 1999), and the DNA-binding HD (Figs. 4A,F). Collectively, *in vitro* calpain-2 cleavage assays and mass spectrometry analysis experimentally validated our hypothesis that MEIS2 is a direct calpain-2 target and suggest that cleavage leads to the separation of functionally important protein domains.

### Phosphorylation determines the sensitivity of MEIS2 towards calpain-2-mediated proteolysis

**Fig. 5.**
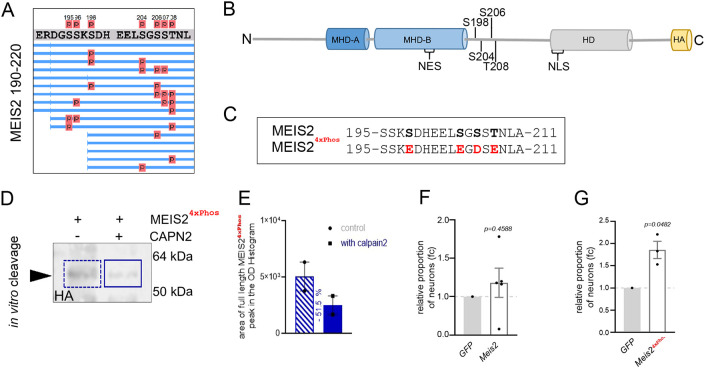
**Phosphorylation within the MHD-B proximal cluster reduces calpain-2-mediated cleavage of MEIS2.** (A) Phosphorylated amino acids in MEIS2 identified by mass spectrometry, focusing on positions 190–220 (see also Fig. S3). Lines represent individual peptides that were detected, red spheres indicate phosphorylated residues. (B) Schematic presentation of MEIS2 protein domain structure. Conserved domains and phosphosites are indicated. (C) Phospho-mimetic MEIS2^4xPhos^–HA construct generated by replacing S198, S204, T208 with aspartic acid and S206 with glutamic acid. (D) Representative immunoblots of MEIS2^4xPhos^–HA following incubation with active calpain-2 (solid blue box) or control without calpain-2 (dashed blue box). Immunoblot for HA epitope. Arrowhead, respective full-length proteins detected by HA-specific antibodies. Sizes of marker protein bands are indicated on the right. (E) Quantification of the results in D (mean±s.e.m.; *n*=2). Quantification of full-length MEIS2 signal on western blot following incubation with calpain-2 or control without calpain-2. For quantification area of full-length MEIS2 peak on optical density (OD) histogram of the respective lane was analyzed. (F,G) Number of DCX+ neurons following overexpression of MEIS2-HA (F, *n*=5) or MEIS2^4xPhos^-HA (G, *n*=3), expressed as fc relative to empty viral backbone control (GFP). Data are represented as mean±s.e.m. *P*-value is indicated (Wilcoxon one sample *t*-test). *n*=biological replicates.

**Fig. 6.**
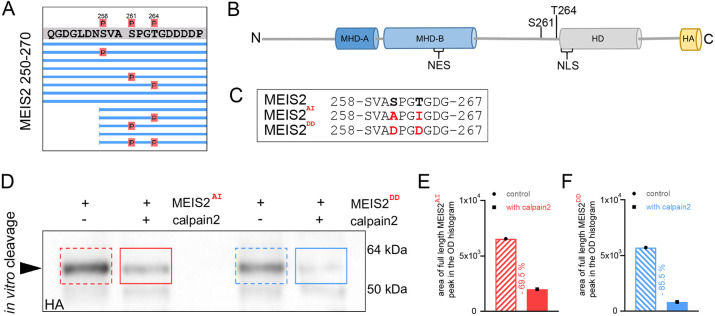
**Phosphorylation within the PEST-sequence enhances cleavage of MEIS2 by calpain.** (A) Phosphorylated amino acids in MEIS2 identified by mass spectrometry, focusing on positions 250–270 (see also Fig. S3). Lines represent individual peptides that were detected; red spheres indicate phosphorylated residues. (B) Schematic presentation of MEIS2 protein domain structure. Conserved domains and the phosphosites at S261 and T264 are highlighted. (C) Partial amino acid sequence of MEIS2 showing S261, T264 and surrounding residues. Phospho-mimetic MEIS2^DD^ construct generated by replacing S261 and T264 with glutamic acid. Non-phospho-mimetic MEIS2^AI^ construct generated by replacing S261 by alanine and T264 by isoleucine. (D) Representative immunoblot images of MEIS2^DD^–HA (blue, right) or MEIS2^AI^–HA (red, left) after incubation with active calpain-2 (solid boxes) or control without calpain-2 (dashed boxes). Immunoblot for HA epitope. Arrowhead, respective full-length proteins detected by HA-specific antibodies. Sizes of marker protein bands are indicated on the right. (E,F) Quantification of full-length MEIS2 signal for the western blot shown in D following incubation with calpain-2 (solid bar) or control without calpain-2 (striped bar). For quantification area of full-length MEIS2 peak on optical density (OD) histogram of the respective lane was analyzed.

Because calpain proteases recognize the three-dimensional structure of their substrates, substrate recognition and proteolytic cleavage by calpains are sensitive to conformational change of the target protein. Protein phosphorylation is a common PTM that locally changes electrochemical properties of a protein and thereby modulates its conformation. We therefore considered the possibility that the ability of calpain-2 to process MEIS2 might depend on the phosphorylation state of MEIS2. Phosphoproteomic analysis by mass spectrometry of MEIS2, which was ectopically overexpressed and purified from HEK 293T cells, identified two clusters of phosphorylated amino acids. First, an MHD-B-proximal cluster comprised positions S195, S196, S198, S204, S206, S207 and T208. Of these six sites, phosphorylation at up to four residues could be observed simultaneously, most often involving S198, S204, S206 and T208 (Fig. 5A,B; Fig. S3). Second, we noticed a smaller, HD-proximal cluster comprising S258, S261 and T264, of which phosphorylation predominately occurred at S261 and T264 (Fig. 6A,B; Fig. S3). Notably, the MHD-B-proximal phosphosites overlap with the cleavage sites identified in our first mass spectrometry analyses (Fig. 4F). We replaced S198, S204, S206 and T208 by phospho-mimicking amino acid substitutions to generate MEIS2^4xPhos^–HA (Fig. 5C). MEIS2^4xPhos^ was more refractory to cleavage when incubated with native porcine calpain-2 than with wild-type MEIS2 (Fig. 5D,E). In addition, retroviral overexpression of MEIS2^4xPhos^, but not wild-type MEIS2 lacking the phospho-mimetic amino acids at the MHD-B-proximal cluster, significantly increased the number of DCX+ neurons after short term *in vitro* differentiation (Fig. 5F,G). Thus, clustered phosphorylation near the MHD-B proximal cleavage site counteracts calpain-2-mediated fragmentation of MEIS2, and overexpression of a phospho-mimetic (and thus more cleavage-resistant) MEIS2 variant in aNS enhances neurogenesis.

The HD-proximal phosphosites are located within a predicted PEST sequence (Fig. 6A,B; Figs S3 and S4). PEST sequences are rich in proline (P), glutamic acid (E), serine (S) and threonine (T) and have been widely associated with calpain-mediated cleavage and protein degradation (Shumway et al., 1999). Phosphorylation within PEST sequences can enhance fragmentation by calpains (Martinez et al., 2003; Masilamani et al., 2022). Replacing S261 and T264 with phosphorylation-mimetic amino acids to generate MEIS2^DD^ made the resulting protein more sensitive towards cleavage by calpain-2 than by replacing them with uncharged residues (MEIS2^AI^; Fig. 6C–F). Phosphorylation within the PEST sequence thus appears to enhance the already negative charge of this protein domain to increase the accessibility of MEIS2 for calpain-2. Collectively, these results show that the susceptibility of MEIS2 towards cleavage by calpain-2 is regulated by phosphorylation within the stretch of amino acids that links the MHD-B and HD domains, whereby phosphorylation near the MHD-B domain thwarts and phosphorylation within the HD-proximal PEST domain favors proteolysis.

### Dimerization with PBX1 reduces calpain-2-mediated cleavage of MEIS2

**Fig. 7.**
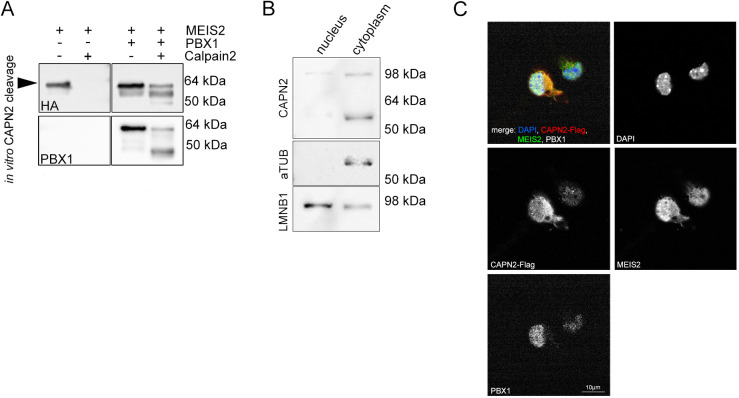
**Dimerization with PBX1 reduces calpain-2-mediated cleavage of MEIS2.** (A) Immunoblot images of calpain-2 cleavage assays of MEIS2-HA (upper-left panel) or co-purified (MEIS2–HA)–PBX1 dimers (upper-right panel), stained for HA. Lower panels: membrane re-probed for PBX1. Arrowhead, full-length MEIS2–HA. (B) Immunoblot of nuclear and cytoplasmic extracts isolated from aNS and serially developed for the antigens listed on the left. Immunoreactivity for CAPN2 was primarily detected in the cytosolic fraction. α-tubulin (aTUB) and lamin B1 (LMNB1) served are reference for the quality of subcellular fractionation. (C) A single confocal image of aNS transduced with Flag-tagged CAPN2 and stained with antibodies directed the Flag-epitope, MEIS2 and PBX1, counterstained with DAPI. Individual channels are shown in grayscale for clarity. MEIS2 immunoreactivity in the cytoplasm overlaps with CAPN2 but not with PBX1. Misexpression of CAPN2–Flag for detection was necessary because the CAPN2-specific antibody used in A does not perform well in fluorescent immunocytochemistry. Scale bar: 10 μm. Images in this figure representative of three repeats.

MEIS family proteins usually exert their biological function as heterodimers with members of the related family of pre-B-cell leukemia homeobox (PBX) transcription factors (Knoepfler et al., 1997). The primary MEIS2 dimerization partner in aNS is PBX1, a protein that participates in multiple steps of adult OB neurogenesis (Grebbin et al., 2016; Remesal et al., 2020). Contact between MEIS with a PBX heterodimerization partner is mediated by the MHD-B domain of MEIS family protein (Bruckmann et al., 2020; Knoepfler et al., 1997; Ryoo and Mann, 1999). Because the site of calpain-2 cleavage we had mapped is located in close proximity to the protein domain involved in MEIS2–PBX1 binding (MHD-B domain; Fig. 4F), we considered the possibility that PBX1 might mask the calpain-cleavage site in the heterodimeric conformation with MEIS2. To test this, MEIS2–HA was purified by immunoprecipitation from overexpressed proteins in HEK 293T cells as either monomers or MEIS2-HA and PBX1 heterodimers and subsequently challenged with native porcine calpain-2. Whereas monomeric MEIS2–HA was virtually undetectable after 30 min of incubation with active calpain-2, MEIS2–HA isolated together with PBX1 largely sustained this treatment (Fig. 7A, top panel). Upon binding to PBX1, MEIS2 thus likely adopts a conformation that protects it from fragmentation by calpain-2. We noted that PBX1 was also sensitive to cleavage by calpain in our assay (Fig. 7A, lower panel). However, PBX1 is nuclear in aNS, whereas CAPN2 and MEIS2 are present in the cytosol (Fig. 7B,C) (Grebbin et al., 2016; Kolb et al., 2018; Storr et al., 2011). MEIS2 proteolysis by calpain-2 thus occurs in a cellular compartment separate from PBX1.

## DISCUSSION

**Fig. 8.**
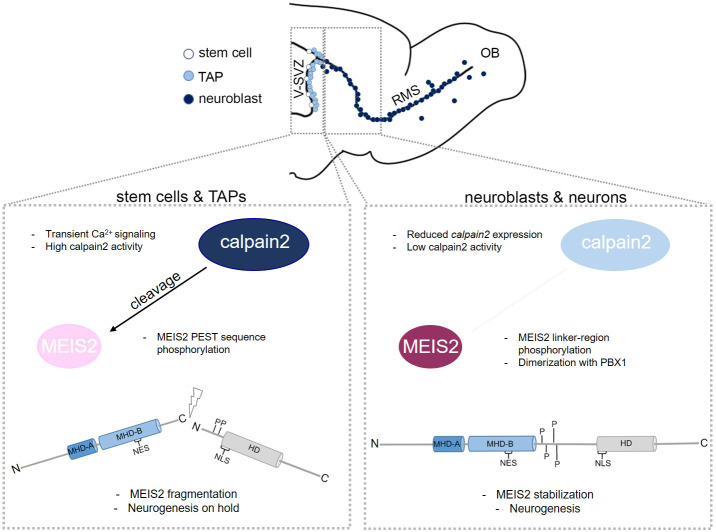
**Schematic overview of MEIS2 post-translational regulation during aNS differentiation.** In the adult V-SVZ, the intracellular protease calpain-2 displays high activity in stem and transient-amplifying progenitor cells (TAP), potentially mediated by transient Ca^2+^ signaling. MEIS2 is a direct target of calpain-2 in aNS. Phosphorylation within a conserved PEST sequence of MEIS2 increases MEIS2 susceptibility for calpain-2 cleavage resulting in MEIS2 fragmentation. The cells remain in an undifferentiated state and neurogenesis is ‘on hold’. During the process of neuronal differentiation, calpain-2 expression and activity decreases and phosphorylation of MEIS2 at conserved serine/threonine residues or the dimerization with PBX1 reduces the sensitivity of MEIS2 towards cleavage by calpain-2. MEIS2 is stabilized and contributes to olfactory bulb (OB) neurogenesis.

The process of generating new neurons from resident stem cells in the adult brain has been extensively studied at the genetic, epigenetic and transcriptional level, but the proteome as a regulatory entity in this process has only recently become a focus of interest (Kjell et al., 2020). Here, we report a new mechanism of post-translational regulation of adult neurogenic differentiation through cleavage of the transcription factor MEIS2 by the intracellular Ca^2+^-dependent protease calpain-2. We show that this cleavage is controlled by phosphorylation at two clusters within the MEIS2 protein, phosphorylation of which affects the stability of MEIS2 in opposite ways, as well as by heterodimer formation between MEIS2 and PBX1. Notably, modulating calpain-2 activity levels affected neurogenic differentiation from sphere-forming cells of the adult V-SVZ. Our findings hence reveal a novel form of control over adult neurogenesis, namely by proteolytic cleavage of a neuronal fate determinant. Given the dependence of calpain-2 on the intracellular Ca^2+^ concentration, our results also provide a possible explanation for how adult V-SVZ neurogenesis might be controlled by Ca^2+^ dynamics (Fig. 8).

### Involvement of Calpain-2 and Ca^2+^ dynamics in the regulation of adult V-SVZ neurogenesis

Calpain-2 expression and proteolytic activity is dynamically regulated when adult V-SVZ stem cells progress towards neurogenesis. Calpain activity is high in undifferentiated cells but declines rapidly upon neuronal differentiation, a process that is accompanied by increased stability of MEIS2. The endogenous calpain inhibitor CAST co-exists with calpains in most cell types, suggesting a pivotal role of this inhibitor in the regulation of calpain activity (Wendt et al., 2004). Indeed, mice mutant for *Cast* (*Cast^−/−^*) exhibited reduced numbers of migrating, DCX+ neuroblasts in the V-SVZ and RMS (Machado et al., 2015). However, *Cast* was barely detectable in aNS and neurons in our assay. These low levels of *Cast* in the progeny of V-SVZ-derived adult neural stem cells together with the fact that deletion of *Cast* was not targeted to stem- or progenitor cells in the *Cast^−/−^* mouse model suggest that the decreased neurogenesis seen in these animals likely does not stem from a defect that arises from the stem cells themselves but rather from another cellular compartment of the V-SVZ stem cell niche.

Calpain proteases are tightly regulated by short-term and long-term acting factors. Expression of CAST or the availability of CAPN4 (CAPNS1) affect calpain activity in periods of hours to days, whereas fluctuations in intracellular Ca^2+^ or PTM in response to extracellular signals affect calpain catalytic activity immediately. We treated aNS with pharmacological agents known to induce neuronal differentiation in V-SVZ progenitor cells *in vivo* or *in vitro*. This treatment significantly reduced calpain cleavage activity, suggesting that calpain activity in these cells is sensitive to stem cell niche signaling pathways. This observation not only links calpain activity to cues from the stem cell niche, but it is also in line with other studies showing that extracellular signal-regulated kinase (ERK) signaling downstream of the EGF receptor activates calpain-2 (Glading et al., 2004).

Changes in the intracellular Ca^2+^ concentration affect the bipartite structure of the calpain protease domain. Without Ca^2+^, the active-site residues and potential substrate-binding cleft remain in a non-functional conformation (Hosfield, 1999; Strobl et al., 2000). Ca^2+^ binding activates calpain by causing dynamic conformational changes sufficient to fuse the two protease core domains into a functional protease domain (Moldoveanu et al., 2002). The required Ca^2+^ concentration for *in vitro* activation of calpain-2 is in the high micromolar to low millimolar range (Moldoveanu et al., 2002). Given that the intracellular Ca^2+^ concentration is normally around 100 nM (Clapham, 1995), which is insufficient for calpain-2 activation, enzymatic activity must be achieved by Ca^2+^ influx from the extracellular space or release from intracellular Ca^2+^ stores. Transcriptomic analyses and Ca^2+^ imaging have established that the V-SVZ is a region with particularly prominent Ca^2+^ dynamics (Beckervordersandforth et al., 2011; Kraft et al., 2017; Lacar et al., 2011). Interestingly, Ca^2+^ dynamics influence stem cell behavior. For instance, fluid shear stress of the cerebral spinal fluid initiates Ca^2+^ influx in quiescent stem cells residing in the V-SVZ to modulate stem cell proliferation, survival and neurogenesis (Petrik et al., 2018). Likewise, quiescent and activated NSCs possess distinct Ca^2+^ signatures, and several micro-environmental signals converge on intracellular Ca^2+^ pathways to regulate V-SVZ NSC quiescence and activation in response to the day–night cycle (Gengatharan et al., 2021). Our discovery of calpain-2-dependent control over the neuronal transcription factor MEIS2 now provides a mechanistic explanation for how changes in intracellular Ca^2+^ concentrations in the V-SVZ neurogenic system can be translated into a transcriptional output and altered neurogenic gene expression.

### MEIS2 cleavage by calpain-2 is subject to intricate regulation

Phosphorylation is a widespread and diverse PTM that within milliseconds changes the electrochemical properties of a protein and thereby its conformation. Phosphorylation is therefore a simple mechanism by which the sensitivity of a potential substrate towards calpain can be modulated in response to intracellular signaling cascades and cell extrinsic cues. Because phosphoproteomic experiments on endogenous MEIS2 protein in adult V-SVZ stem and progenitor cells is limited by its low expression and protein stability in these cells, we here mapped phosphosites on ectopically expressed purified MEIS2 protein. Phosphoproteomic analyses of purified MEIS2 identified two clusters of phosphorylated amino acids, located at either end of an unstructured domain that serves as a linker between the MHD-A and MHD-B domain, through which MEIS2 interacts with its heterodimerization partner PBX1 and the DNA-binding HD (Bruckmann et al., 2020; Geerts et al., 2005). Phosphosites in both clusters lie within local accumulations of aspartate and glutamate residues, amino acids with electrically charged acidic side chains. Phosphorylation at these positions is therefore expected to enhance the negative charge that already exists locally at these sites. Unexpectedly, phosphorylation at these two domains affected the sensitivity of MEIS2 towards calpain-2 in opposite ways – phosphorylation at the MHD-B-proximal cluster made the protein more resistant to calpain-2 protease, whereas phosphorylation within the HD-proximal PEST sequence enhanced cleavage by calpain-2. The latter observation is consistent with published reports about calpain-mediated processing of other PEST-domain-containing proteins (Martinez et al., 2003; Masilamani et al., 2022). A study on primary hematopoietic stem and precursor cells recently reported that phosphorylation of MEIS1 at corresponding sites, including at S196, affects the stability of MEIS1 (Garcia-Cuellar et al., 2022). Although the protease(s) involved in MEIS1 proteolysis were not identified by the authors, their results argue that phosphorylation within the MHD-B-proximal cluster is more generally involved in the regulation of MEIS protein integrity and activity. In this context it is noteworthy that accumulation of possible phosphosites between the MHD-B and HD domains is conserved in vertebrate MEIS1 and MEIS2 and their invertebrate homolog homothorax (hth) (Reichlmeir et al., 2021) (Fig. S4). Calpain-2-mediated cleavage and its modulation by phosphorylation might thus be a universal feature of MEIS proteins across the animal kingdom.

Explorative analysis into the potential kinases responsible for the distinct phosphorylation of MEIS2 during neuronal differentiation by the kinase prediction tool NetPhos3.1 (Blom et al., 1999) implicated phosphorylation sites S198 and T264 as potential target sites for casein kinase 2 (CK2; CSNK2A1), whereas phosphorylation at position S261 might be mediated by CK2, casein kinase 1 (CK1; CSNK1A1), or mitogen-activated protein kinase (p38MAPK). Notably, both MAPK and CK2 are recognized downstream targets of the EGFR signaling pathway. Ji and colleagues, for instance, reported that EGFR directly interacts with CK2 in cancer cell lines, resulting in enhanced CK2 activity and α-catenin phosphorylation (Ji et al., 2009), whereas a study from our laboratory showed that pharmacological EGFR-inhibition of aNS by AG-1478 initiates nuclear accumulation of MEIS2 and neuronal differentiation even under culture conditions that otherwise keep cells in an undifferentiated cellular state (Kolb et al., 2018). Yet, how EGFR signaling might act through CK2 to influence the phosphorylation status of MEIS2 and subsequent maturation of aNS towards neurogenesis remains a complex issue. This complexity is underscored by the fact that T264 phosphorylation enhanced and S198 phosphorylation dampened the susceptibility of MEIS2 towards calpain cleavage in our assays, even though computer prediction links both phosphosites to EGFR signaling. In conclusion, the intricate relationship between kinase phosphorylation and its potential effects on the stability or degradation of endogenous MEIS2 protein requires further investigation.

Mass spectrometry analysis of the MEIS2 protein fragments showed that calpain-2 separates the MHD-A/B and HD domains. As shown for the splice isoform MEIS2D, the MEIS2 C-terminus folds back onto the MHD-A/B domain in monomeric MEIS2, inducing an autoinhibitory conformational constraint on the MEIS2 transcriptional activity that is relieved by MEIS–PBX dimer formation (Hyman-Walsh et al., 2010). It is intriguing to propose that this conformational change might also determine the vulnerability of MEIS2 to calpain. In this context, it is worth mentioning that PBX1 is nuclear in aNS, whereas CAPN2 is predominantly cytoplasmic and MEIS2 is actively exported from the nucleus to the cytoplasm as a means to avoid uncontrolled neuronal differentiation (Fig. 7C) (Grebbin et al., 2016; Kolb et al., 2018; Storr et al., 2011). This subcellular compartmentalization should ensure that proteolysis of MEIS2 by calpain-2 occurs primarily outside the cell nucleus, thus separating the site of MEIS2 transcriptional activity from the site of its cleavage. Considering that association of MEIS with PBX also masks the nuclear export signal in MEIS2 (Kolb et al., 2018), MEIS–PBX heterodimerization seems to serve three purposes at once: it prevents cleavage of MEIS2 by calpain, breaks the cycle of its nuclear export to facilitate MEIS2 accumulation in the cell nucleus and relieves the MEIS2 transcriptional activation domain from auto-inhibition.

Together with published work, the results presented here allow us to sketch a model of how multilevel regulation of MEIS2 contributes to adult V-SVZ neurogenesis. *Meis2* transcripts are already present in quiescent NSCs (Beckervordersandforth et al., 2010), yet translation is prevented by the activity of miR-204 (Lepko et al., 2019). MiR-204 is delivered by extracellular vesicles from the choroid plexus and is sensed by quiescent stem cells through their primary cilium, which exposes them to signals in the cerebrospinal fluid (Lepko et al., 2019). Inhibition of MEIS2 protein production by miR-204 is relieved when cells progress towards activated NSCs and TAPs, but CRM-mediated nuclear export continues to prevent MEIS2 accumulation in the nucleus of these cells (Kolb et al., 2018), leaving it cytoplasmic and susceptible to cleavage by calpain-2 (this study). In this way, high calpain enzymatic activity in adult V-SVZ-derived progenitor cells counteracts precocious neuronal differentiation. The rapid drop in calpain activity that occurs when cells start to differentiate, likely together with altered phosphorylation of MEIS2 then stabilize MEIS2 (this study). In parallel, methylation on a conserved arginine residue abolishes association of MEIS2 with the nuclear export machinery and allows it to translocate to the nucleus (Kolb et al., 2018). There, MEIS2 dimerizes with PBX1, participates in the activation of downstream genes and facilitates the progression of adult V-SVZ progenitor cells towards neurogenic differentiation (Hau et al., 2017; Kolb et al., 2018). This intricate, multilayer regulation of MEIS2 in the adult V-SVZ stem cell niche helps to prevent untimely activation of quiescent stem cells as well as the premature and uncontrolled neuronal differentiation of their progeny. By serving as a ‘hub’ to integrate multiple signals, MEIS2 thus likely helps adult V-SVZ stem and progenitor cells to attune their neurogenic output to the physiological state of the individual.

At present, the V-SVZ neurogenic system is the only setting in which the post-transcriptional and post-translational activation of MEIS2 has been studied in such detail and it is therefore not known, whether MEIS2 or other MEIS family members are subject to similarly complex regulatory processes in other physiological or pathophysiological contexts. However, given that MEIS proteins are known oncogenes in leukemias and solid cancers (Collins and Hess, 2016; Schulte and Geerts, 2019), addressing MEIS protein regulation might lead to a deeper understanding of the respective disease processes.

## MATERIALS AND METHODS

### Animals

Sphere-forming cells were isolated from the V-SVZ of 8–10-week-old C57BL/6J mice (RRID:IMSR_JAX:000664), with equal representation of male and female animals. Animals were housed under pathogen-free conditions at the central animal facility of Goethe University Hospital (ZFE), the animal facility of Max Planck-Institute for Brain Research, or purchased from Charles River Laboratories or Janvier Labs. All experiments involving animals were approved by the animal care committee of Goethe University Hospital, Frankfurt, and the government of Hesse, are in accordance with German and EU regulations, and comply with the ARRIVE guidelines.

### Cell culture, neurosphere culture and cellular differentiation assay

HEK 293T cells were purchased from ATCC (CRL 3216), cultured in DMEM high glucose with GlutaMax^TM^ (Invitrogen) supplemented with 10% fetal calf serum (FCS; Invitrogen), and routinely sub-cultivated as 1:10 surface dilution in a new culture dish. Mycoplasma-free status was checked regularly by PCR (AppliChem #A3744).

aNS were isolated and cultivated in in DMEM/F-12 containing 3.5 mM glucose (Gibco), B-27 supplement (Gibco), 20 ng ml^−1^ fibroblast growth factor-2 (FGF2, human recombinant; Peprotech) and 20 ng ml^−1^ epidermal growth factor (EGF, human recombinant; Peprotech) as described previously (Agoston et al., 2014). To generate second passage aNS, primary spheres were dissociated with accutase (PAA) by incubation for 10 min at 37°C and moderate shaking. Dissociated cells were pelleted, washed once and seeded at 1×10^5^−2×10^5^ cells/ml in fresh aNS culture medium. For *in vitro* differentiation, cells were plated on plating on laminin-coated (1 μg/cm^2^; Roche) cell culture dishes in medium as above but lacking EGF and FGF2. To enrich for neurons for calpain-2 activity measurements (Fig. 1B,C), aNS were retrovirally transduced with *Pax6* 48 h prior to differentiation; differentiation was carried out on laminin-coated dishes in medium lacking EGF and FGF2 but supplemented with 20 ng ml^−1^ brain-derived neurotrophic factor (BDNF; human recombinant, Peprotech) for the times indicated. All cells were cultured in a humidified atmosphere at 5% CO2 and 37°C. Inhibitor treatments were performed with 100 mM EGFR inhibitor (AG1278, Selleckchem, S2728), 10 μM MEK1/2 inhibitor (AZD08330, Cayman Chemicals, 869357-68-6), or 1 μM PKC inhibitor (Gö6976, Cayman Chemicals, 136194-77-9) for 12 h. Control cells were treated with the respective amount of DMSO. After 12 h, cells were subjected to the calpain activity assay.

### Calpain activity assay

Cells were pelleted by centrifugation (800 ***g*** for 3 min), resuspended in 20 mM HEPES, 10 mM KCl, 1.5 mM MgCl_2_, 1 mM DTT, 0.1% Triton X-100, and lysed for 30 min at 4°C. A minimum of 10 μg total protein per reaction was diluted to a final volume of 100 μl with calpain activity assay reaction buffer (20 mM HEPES, 10 mM KCl, 1.5 mM MgCl_2_ and 1 mM DTT). Duplicates for each condition were prepared of which one was treated with 100 μM calpain inhibitor calpeptin [CPT (Cayman Chemicals, 119591-20-5), 30 min at room temperature]. Calpain activity was measured as 360 nm and 460 nm (excitation/emission) in 2 mM CaCl_2_, 5 mM cysteine and 50 μM CAPN substrate (N-succinyl-Leu-Leu-Val-Tyr 7-Amido-4-Methylcoumarin; ACM substrate, Sigma Aldrich, S6510) after 1 h incubation at 37°C in a 96-well plate and using an Infinite 2000 plate reader (Tecan). Emission values obtained following CPT treatment were subtracted from emission values obtained from untreated control samples to exclude the contribution of other proteases. Calpain activity was expressed relative to control condition or in total value as relative fluorescence units (RFUs) as indicated in the figure legends.

### Immunoblotting

Whole-cell protein extracts were generate using RIPA buffer (50 mM, Tris-HCl pH 7.5, 150 mM NaCl, 0.1% SDS, 0.5% sodium deoxycholate, 1% NP40) supplemented with protease and phosphatase inhibitor cocktail (Thermo Fisher Scientific).

Isolation of cytosolic and nuclear protein fractions was achieved by the method described previously (Dignam et al., 1983). In brief, aNS cell pellets were re-suspended in cytosolic extraction buffer (10 mM HEPES, 10 mM KCl, 0.1 mM EDTA, 0.1 mM EGTA, 2 mM DTT), incubated for 5 min on ice, IGEPAL was added to a final concentration of 1% followed by centrifugation at 14,000 ***g*** for 1 min at 4°C. The supernatant containing the cytosolic proteins was collected. The nuclear cell pellet was re-suspended in nuclear extraction buffer (10 mM HEPES, 10 mM KCl, 0.1 mM EDTA, 0.1 mM EGTA, 2 mM DTT, 400 mM NaCl, 1% IGEPAL) and incubated for 15 min at 4°C, rotating. Insoluble material was removed by centrifugation at 14,000 ***g***, 5 min, 4°C. Protein concentration was determined by BCA assay (Thermo Fisher Scientific). Proteins were separated by SDS-PAGE and transferred to PVDF membranes (Immobilon, Merck Millipore) following standard procedures (for antibody details and dilutions used refer to Table S1). Uncropped images of western blots are shown in Fig. S5.

### Immunocytochemistry, cell imaging and data analysis

For immunostaining, aNS were dissociated by accutase (PAA) and seeded at a density of 0.5×10^5^ cells per 1 cm^2^ on PDL-coated coverslips in culture medium supplemented with EGF and FGF2, attached to the glass by gentle centrifugation (65 ***g***, 2 min), and fixed in 4% paraformaldehyde (PFA) for 10 min. Differentiated cells, seeded at a density of 2×10^5^ cells per 1 cm^2^ on laminin-coated coverslips in culture medium lacking EGF and FGF but containing BDNF for 24 h, were fixed in 4% PFA for 10 min. Immunohistochemical staining was performed following standard procedures without antigen retrieval with the primary antibodies diluted in 0.5% NGS, 0.01% Tween-20 over night at 4°C and secondary antibody incubation for 1 h at room temperature (antibodies listed in Table S2). Immunofluorescent images were quantified by automated cell counting and analysis in ImageJ/Fiji software using the BioVoxxel Toolbox, except for Fig. 2F, which was counted manually by a researcher who was not aware of the experimental conditions (a colleague not involved in the study encoded the individual experiments and conditions with a random alphanumeric code). Threshold filter algorithms were set to match the fluorescent staining intensities of the antigen/antibody combinations used in the respective experiments. The values are displayed as the mean±s.e.m. fold change relative to the appropriate control condition. For quantification of MEIS2 C- and N-terminal staining intensities, nuclei were detected with help of a script-based analysis in the ImageJ/Fiji software and the BioVoxxel Toolbox. The outlines of the nuclei were selected as ROIs and the fluorescence intensity of these ROIs was measured in the red (MEIS2 C-terminal staining) and green channels (MEIS2 N-terminal staining). Cells grown under differentiating conditions for 24 h were analyzed following a similar protocol as DCX-positive and DCX-negative cells separately. A list of software used is given in Table S6, and full scripts and threshold filter algorithms are available in Table S7.

### Computer-based analyses of calpain cleavage sites

Computer-based prediction of the calpain cleavage sites in murine MEIS2 (isoform 2; UniProt identifier: Q3TYM2) was performed using the open-source software GPS-CCD (group-based prediction system-calpain cleavage detector; http://ccd.biocuckoo.org/; Liu et al., 2011). Prediction of the PEST-sequence in MEIS2 was done with the PESTfind software (https://emboss.bioinformatics.nl/cgi-bin/emboss/epestfind). The PEST score obtained for MEIS2 was +14.58; PEST scores greater than +5 are considered significant.

### Protein immunoprecipitation, calpain cleavage assays and assessment by immunoblotting

C-terminally HA-tagged MEIS2 (MEIS2b isoform) was overexpressed in HEK 293T cells by transfection, immunoprecipitated in calpain lysis buffer (50 mM Tris-HCl pH 7.5, 100 mM NaCl, 0.5 mM DTT, 250 mM glucose, 5% glycerol, 1% Triton X-100) with anti-HA-tag antibodies [HA-probe (F-7) mouse sc-7392 Santa Cruz Biotechnology] pre-coupled to Dynabeads (Invitrogen), and eluted with 5 μg μl^−1^ HA peptide in Tris-buffered saline (TBS) for at least 30 min at 6°C. *In vitro* cleavage was performed by addition of native porcine calpain-2 protease (1.25 μg, Creative BioMart, CAPN2-22P), 5 mM Ca^2+^ and 1 mM cysteine and incubation for 30 min at 37°C. Control samples were treated identically except omitting calpain-2. Glutathione S-transferase (GST) served as negative control. To allow for MEIS2–PBX1 dimer formation, HEK 293T cells were simultaneously transfected with plasmids expressing *Pbx1* and HA-tagged *Meis2b* (Table S5). Protein complexes were isolated by immunoprecipitation with HA-specific antibodies. MEIS2–HA monomers, generated by transfection of *Meis2b*–HA-expressing plasmids alone, served as control. SDS-PAGE and western blot analysis were performed following standard procedures. Densitometric analysis of full-length MEIS2 was performed in ImageJ following published protocols (Stael et al., 2022) as follows: western blots were developed with the LI-COR Odyssey system and LI-COR Image Studio software (version 2.1.10). Default exposure settings were applied to ensure exposure within the linear range. Blot images were opened in ImageJ software to determine the mean gray values of the specific bands. Using the rectangular selections tool from the ImageJ toolbar respective lanes for analyzation were selected. ImageJ software converted the band grayscale into an optic density (OD) histogram, and the area of the full-length MEIS2 peak was the quantified value of the grayscale. The mean gray values of only the full-length MEIS2 band at ∼60 kDa were determined and further analyzed. Subcellular fractionation was performed as previously described (Dignam et al., 1983).

### Profiling of calpain transcript expression

RNA extraction (from aNS and neuron cell pellets) and reverse transcription to cDNA were performed with RNeasy Mini Kit (Qiagen) and RevertAid H Minus First Strand cDNA Synthesis Kit (Thermo Fisher Scientific), respectively. qPCR was undertaken in triplicates with ABsolute qPCR SYBR Green Fluorescein mix (Thermo Fisher Scientific) and the primer pairs listed in Table S3. Analysis was with a Bio-Rad CFX qPCR machine, expression was normalized to levels of β*-*actin (*ACTB*) mRNA following the ΔCq method.

### Constructs for viral transduction and knockdown of aNSCs

shRNA-mediated knockdown of *Capn4* was achieved by pGIPZ vectors (Dharmacon, Thermo Fisher Scientific). Non-silencing shRNAmir RHS4346 served as control. V3LMM_473000, achieving a reduction of *Capn4* transcripts to 39% within 48 h in aNS compared to the RHS4346, was used for further experiments. For *Capn2* or *Capn2^S50D^* overexpression, the respective coding sequences were PCR amplified, outfitted with a N-terminal Flag-tag, sequence verified, and inserted into the MoMLV-derived retroviral vector pSF91 (Hildinger et al., 1999). *Meis2* transgenes are based on murine *Meis2b* (NCBI# CAA04139.1) and were fused to a triple HA tag C-terminally. Point mutations were inserted by site-directed mutagenesis with the primer pairs listed in Table S4. The template DNA was amplified with Phusion DNA-Polymerase (Thermo Fisher Scientific), 5′ ends of the amplicon were phosphorylated by T4 polynucleotide kinase (PNK) for 30 min, followed by heat inactivation of the enzyme and re-ligation overnight. All newly generated mutation constructs were sequence-verified. *Meis2* wild-type and *Meis2^4xPhos^* were inserted into pWPI (Addgene #12254) in front of an IRES–GFP expression cassette, *Meis2* wild-type, *Meis2^DD^* and *Meis2^AI^* into the MoMLV-derived retroviral vector pCLIG, also carrying IRES–GFP (Hojo et al., 2000). All plasmids generated in this study are available from the lead contact upon request, a full list is provided in Table S5. Viral particles were produced by co-transfection of respective transfer vector plasmid with pCMV-VSVG (Addgene #8454) and psPax2 (Addgene #12260) second-generation packaging system for lentivirus production or pCMV-VSVG and pUMVC (Addgene #8449) for retrovirus production. Concentration of viral particles was by ultracentrifugation at 21,000 rpm on a Sorval Surespin rotor at 4°C for 1.5 h. The pelleted viral particles were dissolved in aNS culture medium and used immediately for cell transduction. Viral titers were between 7×10^6^ and 2×10^7^ pfu ml^−1^. For viral infection, aNS were split with accutase, washed once with EGF- and FGF2-containing medium, resuspended in 1 ml EGF- and FGF2-containing medium and incubated for 4 h in the presence of retroviral stocks at 37°C. Cells were then pelleted for 2 min at 800 ***g*** at room temperature, washed once, resuspended in 5 ml EGF- and FGF2-containing medium and allowed to grow under non-adherent conditions until further processed.

### Liquid chromatography-mass spectrometry

To identify phosphorylation sites on MEIS2–HA immunoprecipitates were supplemented with 6 M GdmCl, 50 mM Tris-HCl, 10 mM TCEP and incubated at 95°C for 5 min. Reduced thiols were alkylated with 40 mM chloroacetamid and sample were diluted to obtain a final GdmCl concentration of 0.6 M. Proteins were digested with 1 µg trypsin (sequencing grade, Promega) overnight at 37°C under gentle agitation. Digestion was stopped by adding trifluoroacetic acid to a final concentration of 0.5%. Peptides were eluted in wells of microtiter plates and peptides were dried and resolved in 1% acetonitrile, 0.1% formic acid. Liquid chromatography-mass spectrometry (LC-MS) was performed on a Thermo Fisher Scientific Q Exactive Plus equipped with an ultra-high performance liquid chromatography unit (Thermo Fisher Scientific Dionex Ultimate 3000) and a Nanospray Flex Ion-Source (Thermo Fisher Scientific). Peptides were loaded on a C18 reversed-phase precolumn (Thermo Fisher Scientific) followed by separation on a with 2.4 µm Reprosil C18 resin (Dr. Maisch GmbH) in-house packed picotip emitter tip (diameter 100 µm, 15 cm from New Objectives). PEAKS7 (Bioinformatics Solutions) was used for data analysis. For the analysis of calpain-2-cleaved peptides, MEIS2–HA immunoprecipitates were treated with native porcine calpain-2 protease (1.25 µg, Creative BioMart, CAPN2-22P), 5 mM Ca2+ and 1 mM cysteine and incubation for 30 min at 37°C. Probe preparation was performed as detailed above except the trypsin digestion step was omitted. Calpain-2 cleaved peptides were searched against Meis2–HA without setting of a specific protease. N-terminal acetylation (+42.01), oxidation of methionine (+15.99), deamidation on asparagine, and glutamine (+0.98), carbamidomethylation (+57.02) on cysteine were selected as variable modifications. For the analysis of phosphorylation sites, searches were undertaken against the human reference proteome set (Uniprot, May 2020, 74823 entries) supplemented with murine Meis2–HA limited to semitryptic peptides with at least three misscleavage sites with a false discovery rate (FDR) less than 1%. Variable modifications were carbamidomethylation on cysteine (C,+57.02), phosphorylation on serine and threonine (ST,+79.97) and oxidation on methionine (M, +15.99). Results were uploaded to PRIDE (https://www.ebi.ac.uk/pride/) with the dataset identifier PXD036442.

### Statistical analysis

Statistical analyses were performed using GraphPad Prism version 7.0 (GraphPad, San Diego, California, USA). Unless indicated otherwise, data are visualized as bar graphs (mean±s.e.m.). Numbers of biological replicates, types of controls and statistical tests for each experiment are stated in the figure legends. Lists of primers, antibodies, plasmids and software used can be found in Tables S1–S7.

## Supplementary Material

10.1242/joces.261482_sup1 Supplementary information

## First Person

This article has an associated First Person interview with the first author of the paper.
