# Supplementary material for: The neuronal transcription factor MEIS2 is a calpain-2 protease target
Source: J Cell Sci. 2024 Feb 28;137(4):jcs261482. doi: 10.1242/jcs.261482 (PMC10941658; doi:10.1242/jcs.261482)
Supplement: Supplementary information [file joces-137-261482-s1.pdf]

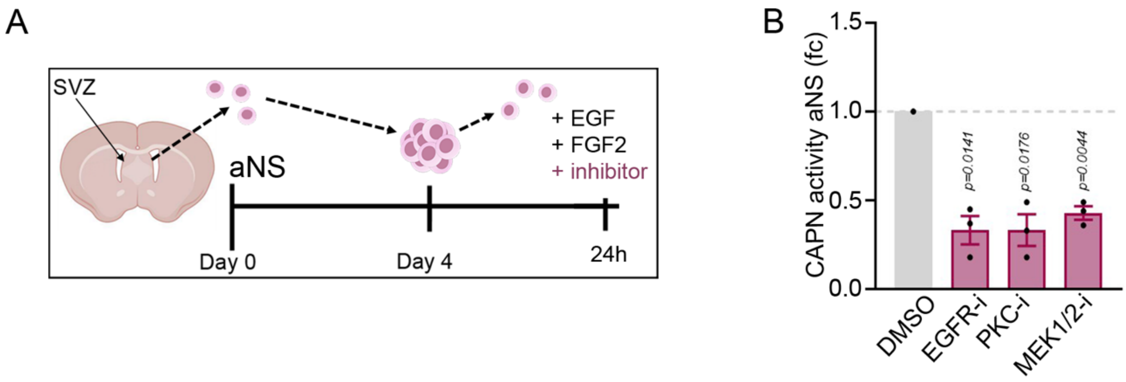

Fig. S1. Treatment with pharmacological agents known to induce neuronal differentiation in aNS causes a net decrease of calpain activity. (A) Schematic outline of the experimental setup. (B) Calpain activity (RFU) in cell extracts derived from aNS incubated with different signaling pathway inhibitors, expressed as fc relative to activity in DMSO treated cells (control). Data were analyzed by one sample t-test. EGFR inhibitor: AG1478, PKC inhibitor: Gö6976, MEK1/2 inhibitor: AZD08330. n=3. P-values are indicated.

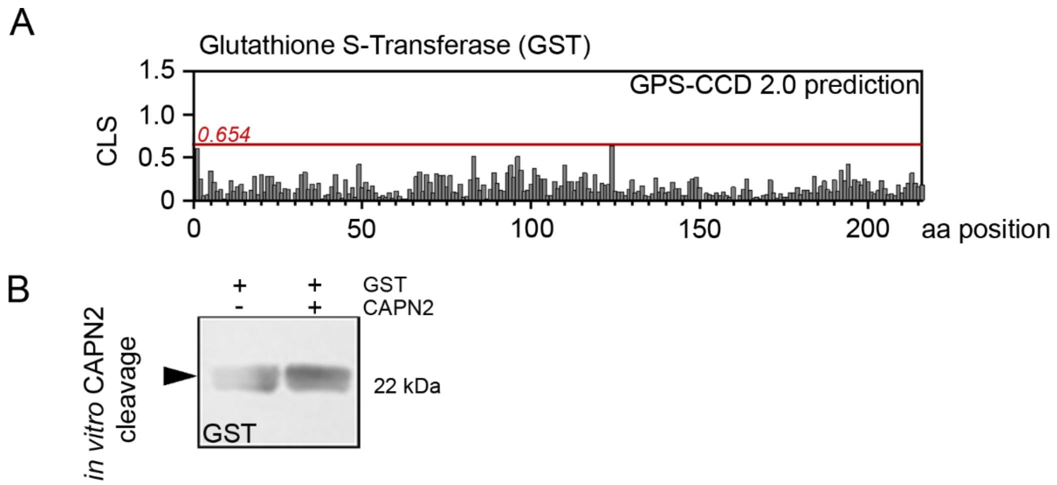

Fig. S2. Glutathione-S-transferase (GST) serves as negative control in calpain2 *in vitro* cleavage assay. (A) Graphical presentation of *in silico* predicted calpain cleavage sites within GST. Predictions were made using the GPS-CCD 2.0 tool. Y-axis represents the cleavage likelihood score (CLS) at respective amino acid position. The red line at prediction score 0.654 corresponds to the maximum default cut-off value above which cleavage is assumed likely. The GST peptide sequence does not show predicted calpain cleavage sites. (B) Representative immunoblot of immuno-purified GST incubated with and without calpain2.

Journal of Cell Science • Supplementary information

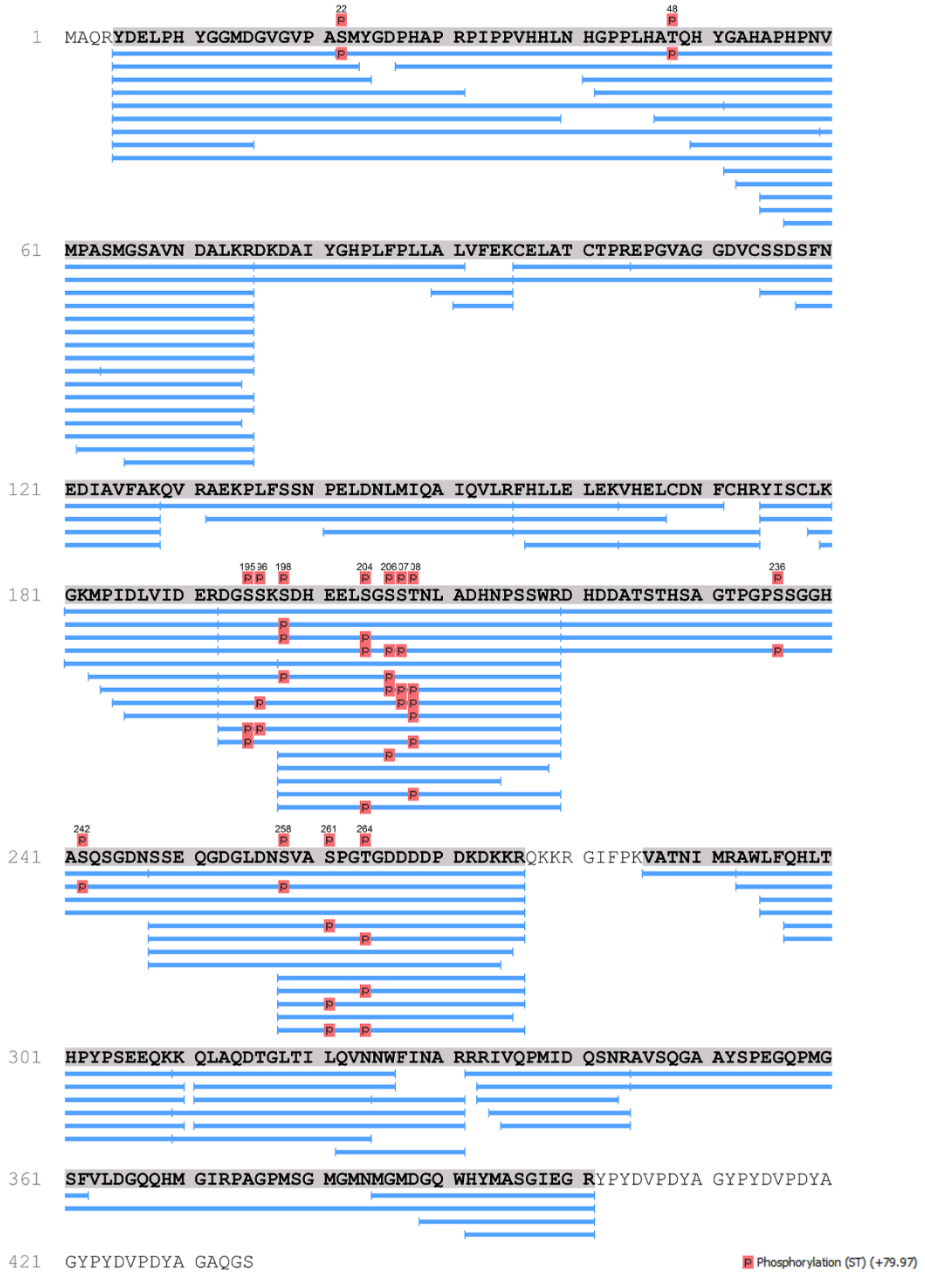

Fig. S3. Graphical summary of sequence coverage of cleavage fragments and location of phosphosites, identified in the present study by mass spectrometry (MS). Blue horizontal lines give a qualitative overview of the different peptides detected from overexpressed MEIS2-HA incubated with calpain2; red spheres mark phosphorylated residues. MEIS2-HA protein is shown as continuous amino acid sequence in single letter code, sequence coverage is highlighted in grey. Given that MS for calpain2 cleavage products did not include a trypsin digestion step during probe preparation, beginning and end of the peptides (marked with small vertical lines) are expected to represent sites cleaved by calpain2. The sequence following amino acid 394 (..WHYM) corresponds to the triple HA-tag.

Journal of Cell Science • Supplementary information

```
msMEIS2     -MAQRYDELPHYGGMDGVGVPASMYGDPHAPRPIPPVHHLN-H-GPPLH----ATQHYGA   53
hsMEIS2     -MAQRYDELPHYGGMDGVGVPASMYGDPHAPRPIPPVHHLN-H-GPPLH----ATQHYGA   53
msMEIS1     -MAQRYDDLPHYGGMDGVGIPSTMYGDPHAARSMQPVHHLN-H-GPPLH----SHQY--P   51
hsMEIS1     -MAQRYDDLPHYGGMDGVGIPSTMYGDPHAARSMQPVHHLN-H-GPPLH----SHQY--P   51
dmHth       MAQPRYDDGLHGYGMDSGAAAAAMYDP-HAGHRPPGLQGLPSHHSPHMTHAAAAAATVGM   59
                ***:  *  ***. .  ::**.  ** :    :: *  * .* :     :

msMEIS2     HA-----------PHPNVMPASMGSAVNDALKRDKDAIYGHPLFPLLALVFEKCELATCT   102
hsMEIS2     HA-----------PHPNVMPASMGSAVNDALKRDKDAIYGHPLFPLLALVFEKCELATCT   102
msMEIS1     HT-----------AHTNAMAPSMGSSVNDALKRDKDAIYGHPLFPLLALIFEKCELATCT   100
hsMEIS1     HT-----------AHTNAMAPSMGSSVNDALKRDKDAIYGHPLFPLLALIFEKCELATCT   100
dmHth       HGYHSGAGGHGTPSHVSPVGNHLMGAIPEVHKRDKDAIYEHPLFPLLALIFEKCELATCT   119
            *             * . :   : .:: :. ******** *********:**********

msMEIS2     PREPGVAGGDVCSSDSFNEDIAVFAKQVRAEKPLFSSNPELDNLMIQAIQVLRFHLLELE   162
hsMEIS2     PREPGVAGGDVCSSDSFNEDIAVFAKQVRAEKPLFSSNPELDNLMIQAIQVLRFHLLELE   162
msMEIS1     PREPGVAGGDVCSSESFNEDIAVFAKQIRAEKPLFSSNPELDNLMIQAIQVLRFHLLELE   160
hsMEIS1     PREPGVAGGDVCSSESFNEDIAVFAKQIRAEKPLFSSNPELDNLMIQAIQVLRFHLLELE   160
dmHth       PREPGVQGGDVCSSESFNEDIAMFSKQIRSQKPYYTADPEVDSLMVQAIQVLRFHLLELE   179
            ****** *******:*******:*:**:*::** ::::**:*.**:**************

msMEIS2     KVHELCDNFCHRYISCLKGKMPIDLVIDERDGSSKSDHEELSGSSTNLAD----------   212
hsMEIS2     KVHELCDNFCHRYISCLKGKMPIDLVIDERDGSSKSDHEELSGSSTNLAD----------   212
msMEIS1     KVHELCDNFCHRYISCLKGKMPIDLVIDDREGGSKSDSEDVTRSAN-L-T----------   208
hsMEIS1     KVHELCDNFCHRYISCLKGKMPIDLVIDDREGGSKSDSEDITRSAN-L-T----------   208
dmHth       KVHELCDNFCHRYISCLKGKMPIDLVIDERDTTKPPELGSANGEGRSNADSTSHTDGAST   239
            ****************************:*:  .  :   . . ..

msMEIS2     ---HNPSS-----WRDHDDATSTHSAGTPGPSSGGHASQS--------------------   244
hsMEIS2     ---HNPSS-----WRDHDDATSTHSAGTPGPSSGGHASQS--------------------   244
msMEIS1     ---DQPSW-----NRDHDDTASTRSGGTPGPSSGGHTSHS--------------------   240
hsMEIS1     ---DQPSW-----NRDHDDTASTRSGGTPGPSSGGHTSHS--------------------   240
dmHth       PDVRPPSSSLSYGGAMNDDARSPGAGSTPGPLSQQPPALDTSDPDGKFLSSLNPSELTYD   299
                 **          :**: *  :..**** *    :  .

msMEIS2     -------------------------------GD--NSSEQGDGLDNSV-ASPGTGDDDDP   270
hsMEIS2     -------------------------------GD--NSSEQGDGLDNSV-ASPGTGDDDDP   270
msMEIS1     -------------------------------GD--NSSEQGDGLDNSV-ASPSTGDDDDP   266
hsMEIS1     -------------------------------GD--NSSEQGDGLDNSV-ASPSTGDDDDP   266
dmHth       GRWCRREWSSPADARNADASRRLYSSVFLGSPDNFGTSASGDASNASIGSGEGTGEEDDD   359
                                           *   .:* .**. : *: :. .**::**

msMEIS2     DKDKKRQKKRGIFPKVATNIMRAWLFQHLTHPYPSEEQKKQLAQDTGLTILQVNNWFINA   330
hsMEIS2     DKDKKRQKKRGIFPKVATNIMRAWLFQHLTHPYPSEEQKKQLAQDTGLTILQVNNWFINA   330
msMEIS1     DKDKKRHKKRGIFPKVATNIMRAWLFQHLTHPYPSEEQKKQLAQDTGLTILQVNNWFINA   326
hsMEIS1     DKDKKRHKKRGIFPKVATNIMRAWLFQHLTHPYPSEEQKKQLAQDTGLTILQVNNWFINA   326
dmHth       ASGKKNQKKRGIFPKVATNILRAWLFQHLTHPYPSEDQKKQLAQDTGLTILQVNNWFINA   419
             ..**.:*************:***************:***********************

msMEIS2     RRRIVQPMIDQSNRAVSQGAAY-SPEGQPMGSFVLDGQQHMGIRPAGPMSGMGMNMGMDG   389
hsMEIS2     RRRIVQPMIDQSNRAVSQGAAY-SPEGQPMGSFVLDGQQHMGIRPAGPMSGMGMNMGMDG   389
msMEIS1     RRRIVQPMIDQSNRAVSQGTPY-NPDGQPMGGFVMDGQQHMGIRAPGLQSMPGEYVARGG   385
hsMEIS1     RRRIVQPMIDQSNRAVSQGTPY-NPDGQPMGGFVMDGQQHMGIRAPGPMSGMGMNMGMEG   385
dmHth       RRRIVQPMIDQSNRAVYTPHPGPSGYGHDAMGYMMDSQAHMMHRPPGDPGF---------   470
            ****************        .  *:    .:::*.* **   *   *   .

msMEIS2     QW-------HYM------------------------------------------------   394
hsMEIS2     QW-------HYM------------------------------------------------   394
msMEIS1     PMGVSMGQPSYTQAQMPPHPAQLRHGPPMHTYIPGHPHHPAVMMHGGQPHPGMPMSASSP   445
hsMEIS1     QW-------HYM------------------------------------------------   390
dmHth       ----HQGYPHYPPAEYYGQHL---------------------------------------   487
                      *

msMEIS2     --------------------             394
hsMEIS2     --------------------             394
msMEIS1     SVLNTGDPTMSAQVMDIHAQ             465
hsMEIS1     --------------------             390
dmHth       --------------------             487
```

Documented phosphorylation sites

MHD-A and MHD-B domain

Homeodomain

Fig. S4. Amino acid alignment of mouse and human MEIS family proteins and D. melanogaster Homothorax (hth). The bipartite MHD domain is shown in red letters, the TALE-HD in blue letters. Phosphosites, either experimentally identified in the present study or documented in literature, are shown with blue background. The alignment was done in Clustal Ω (www.ebi.ac.uk) and the following sequences: mMEIS1: NCBI# NP_001180200.1, mMEIS2: NP_733777, hMEIS1: NP_002389, hMEIS2: AAC529481; summary of phosphosites: Reichlmeir et al., 2021. Hth: FLYBASE FBgn0001235; Phosphosites: https://www.flyrnai.org/tools/iproteindb/web/protein/FBpp0081732/

Journal of Cell Science • Supplementary information

Fig. S5. Blot Transparency

Figure 1E

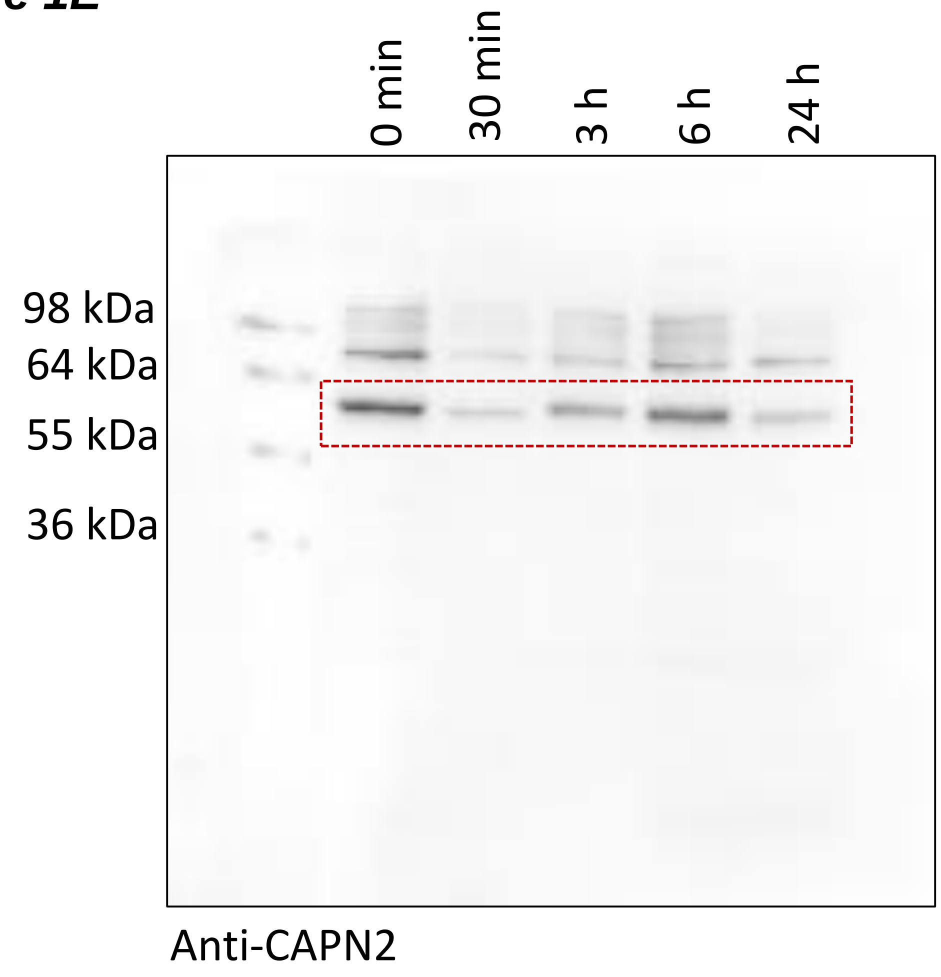

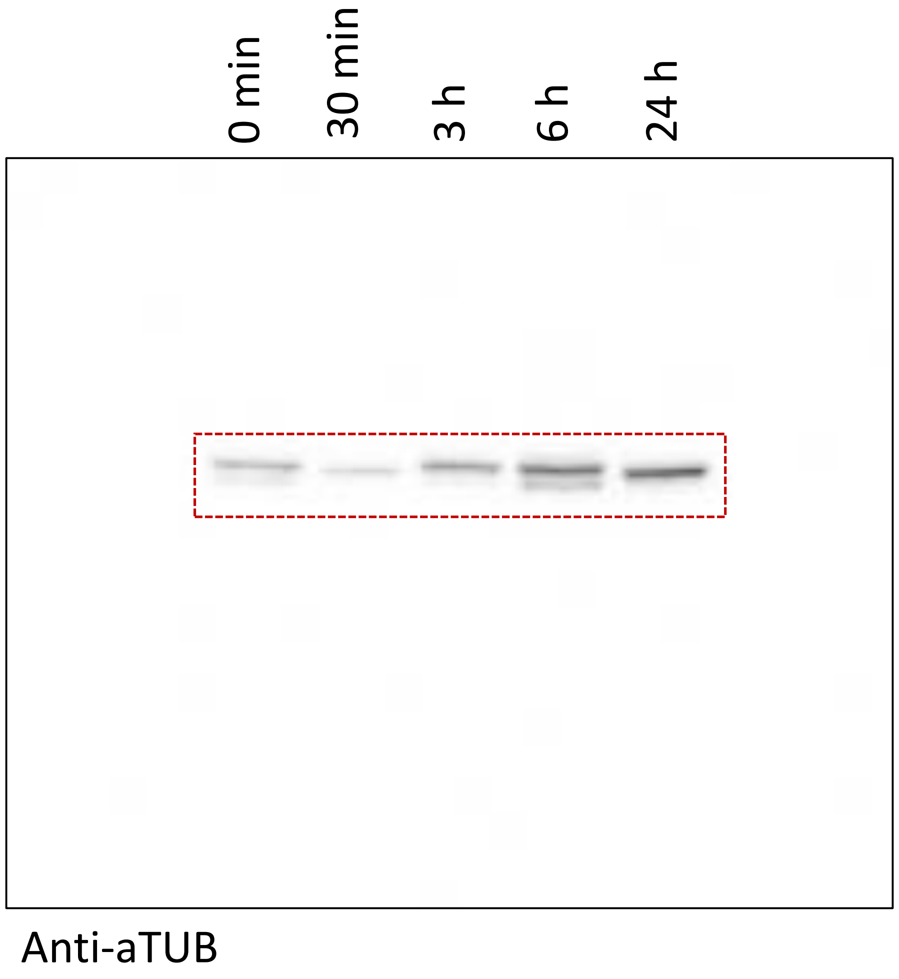

Journal of Cell Science • Supplementary information

Figure 4C

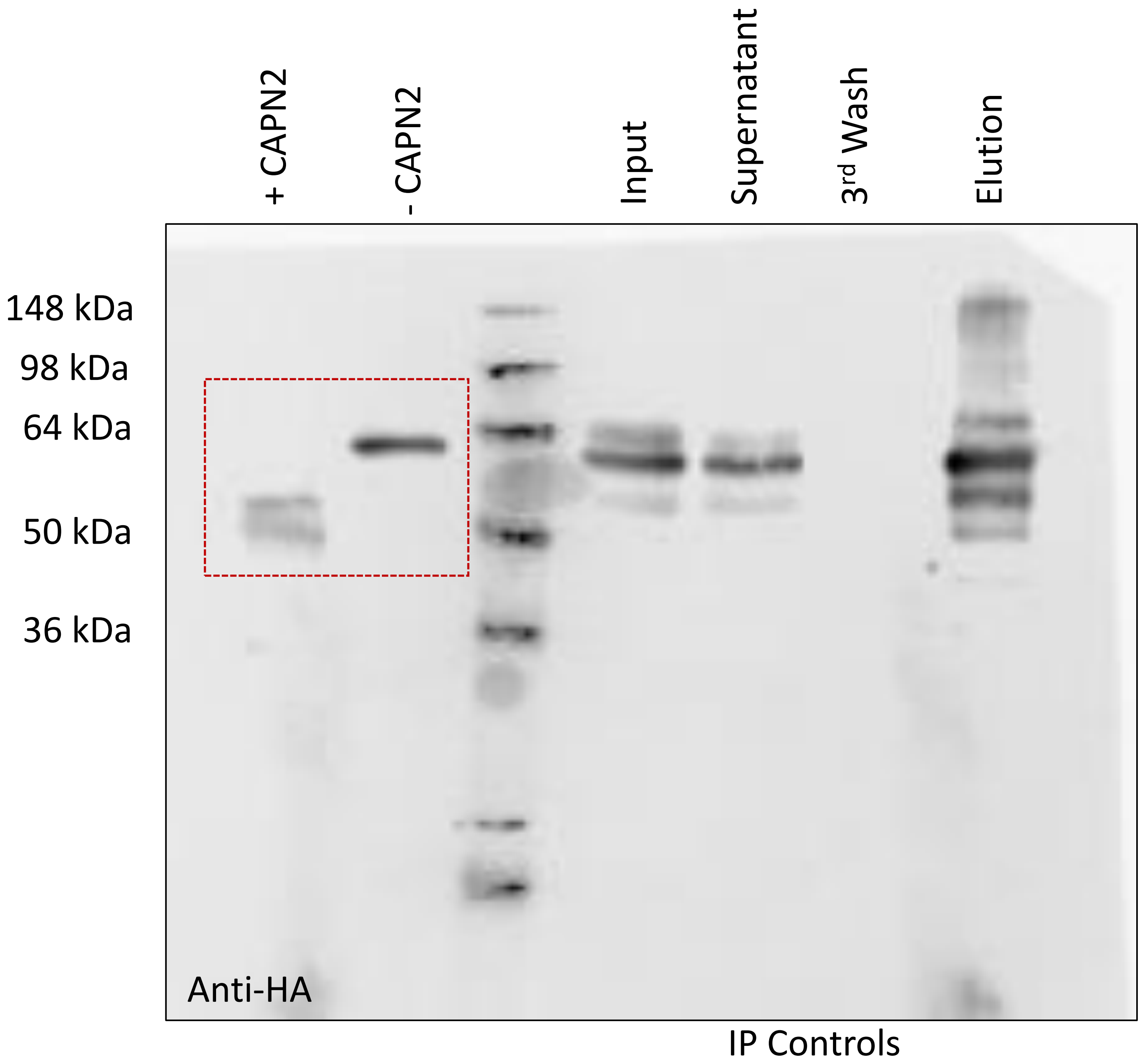

Journal of Cell Science • Supplementary information

Figure 5D

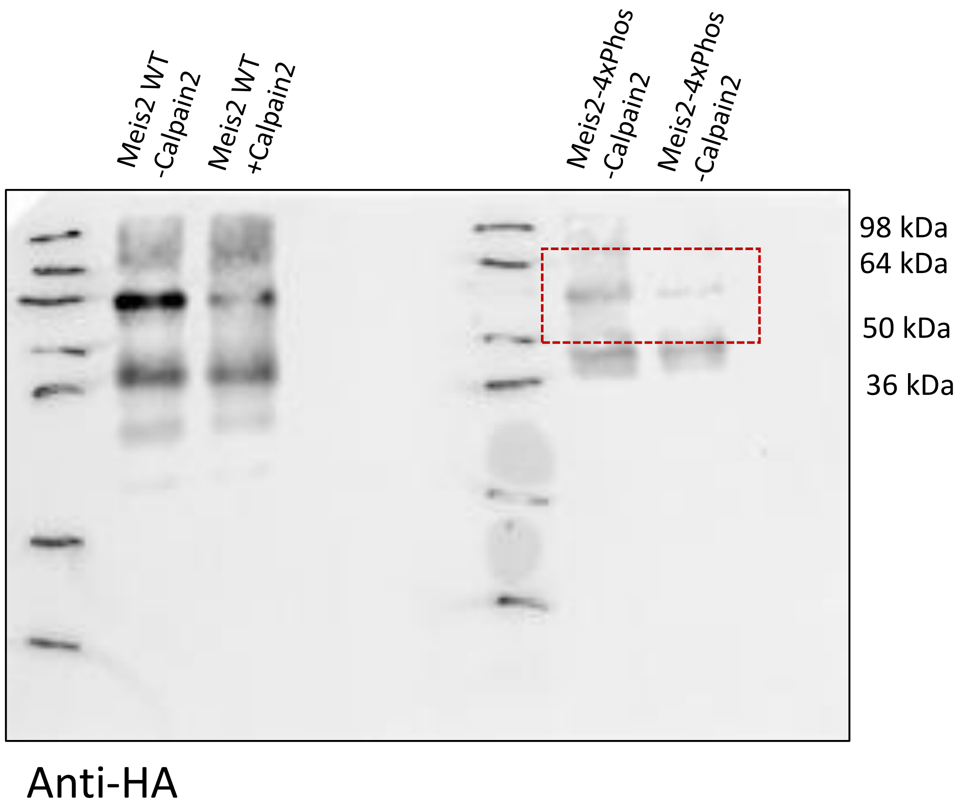

Journal of Cell Science • Supplementary information

Figure 6D

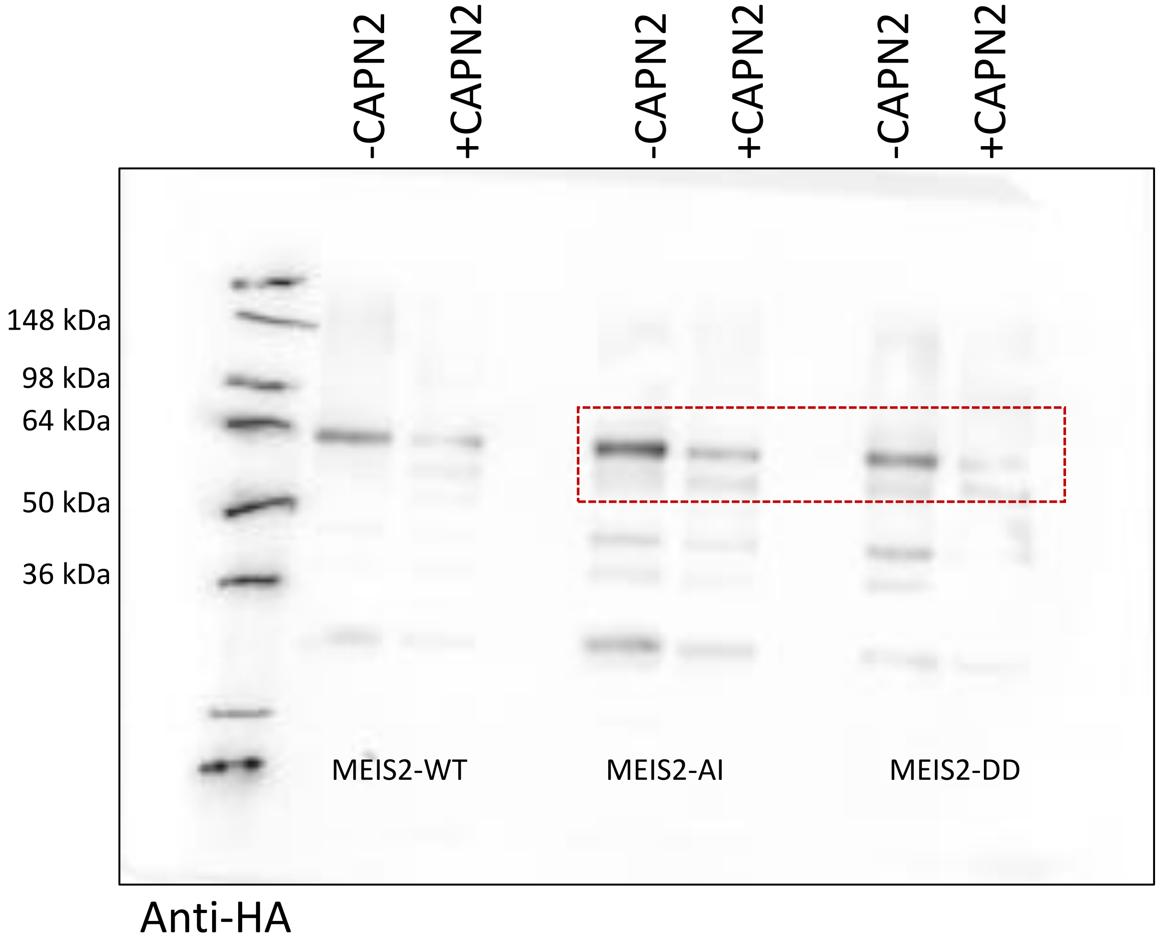

Journal of Cell Science • Supplementary information

Figure 7A

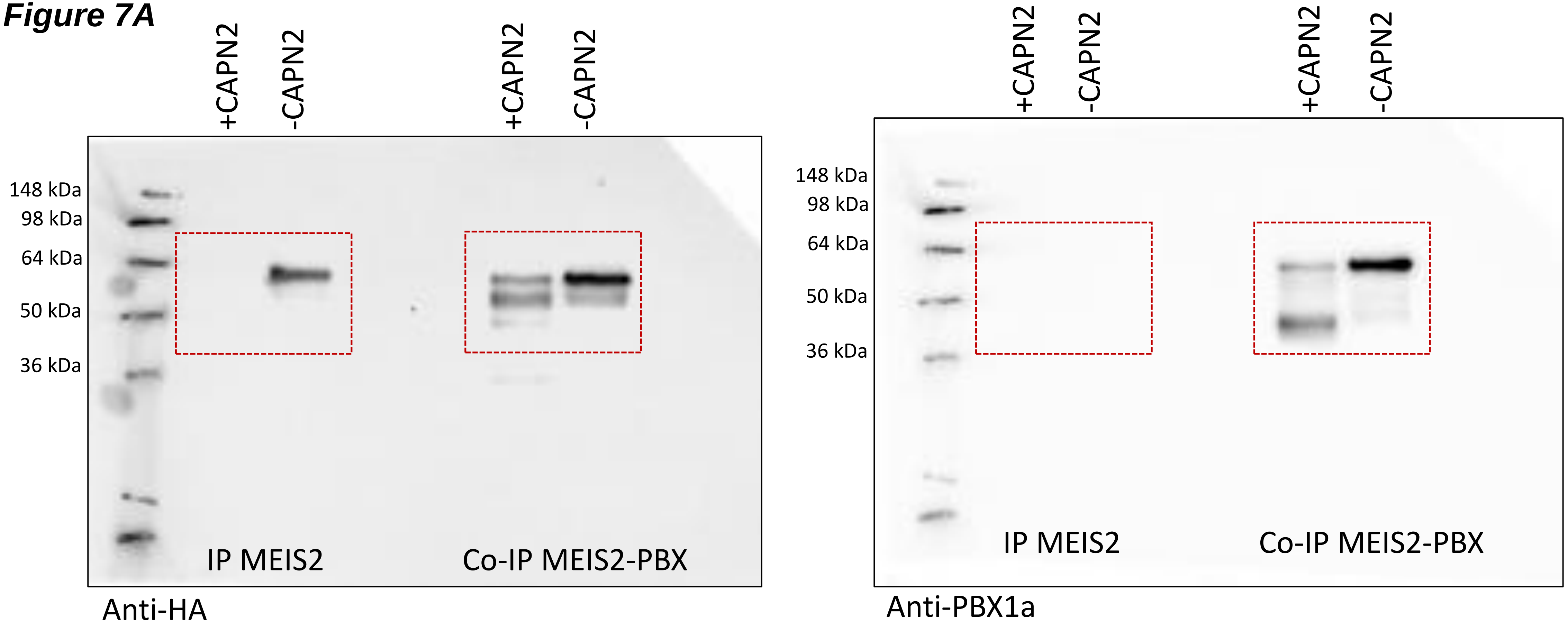
+CAPN2
-CAPN2
+CAPN2
-CAPN2
148 kDa
98 kDa
64 kDa
50 kDa
36 kDa
IP MEIS2
Co-IP MEIS2-PBX
Anti-HA
+CAPN2
-CAPN2
+CAPN2
-CAPN2
148 kDa
98 kDa
64 kDa
50 kDa
36 kDa
IP MEIS2
Co-IP MEIS2-PBX
Anti-PBX1a

Figure 7B

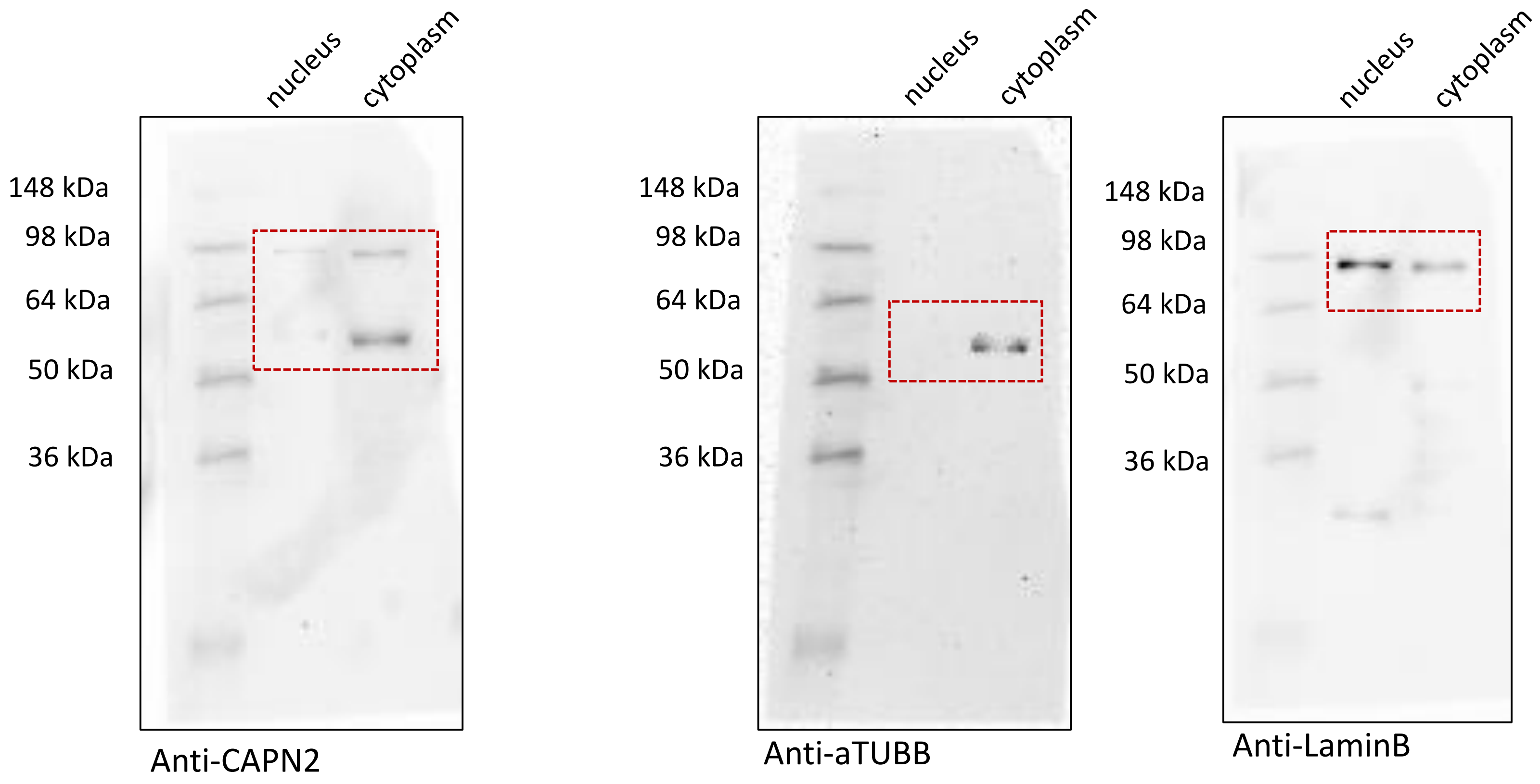
nucleus
cytoplasm
148 kDa
98 kDa
64 kDa
50 kDa
36 kDa
Anti-CAPN2
nucleus
cytoplasm
148 kDa
98 kDa
64 kDa
50 kDa
36 kDa
Anti-aTUBB
nucleus
cytoplasm
148 kDa
98 kDa
64 kDa
50 kDa
36 kDa
Anti-LaminB

Figure S2B

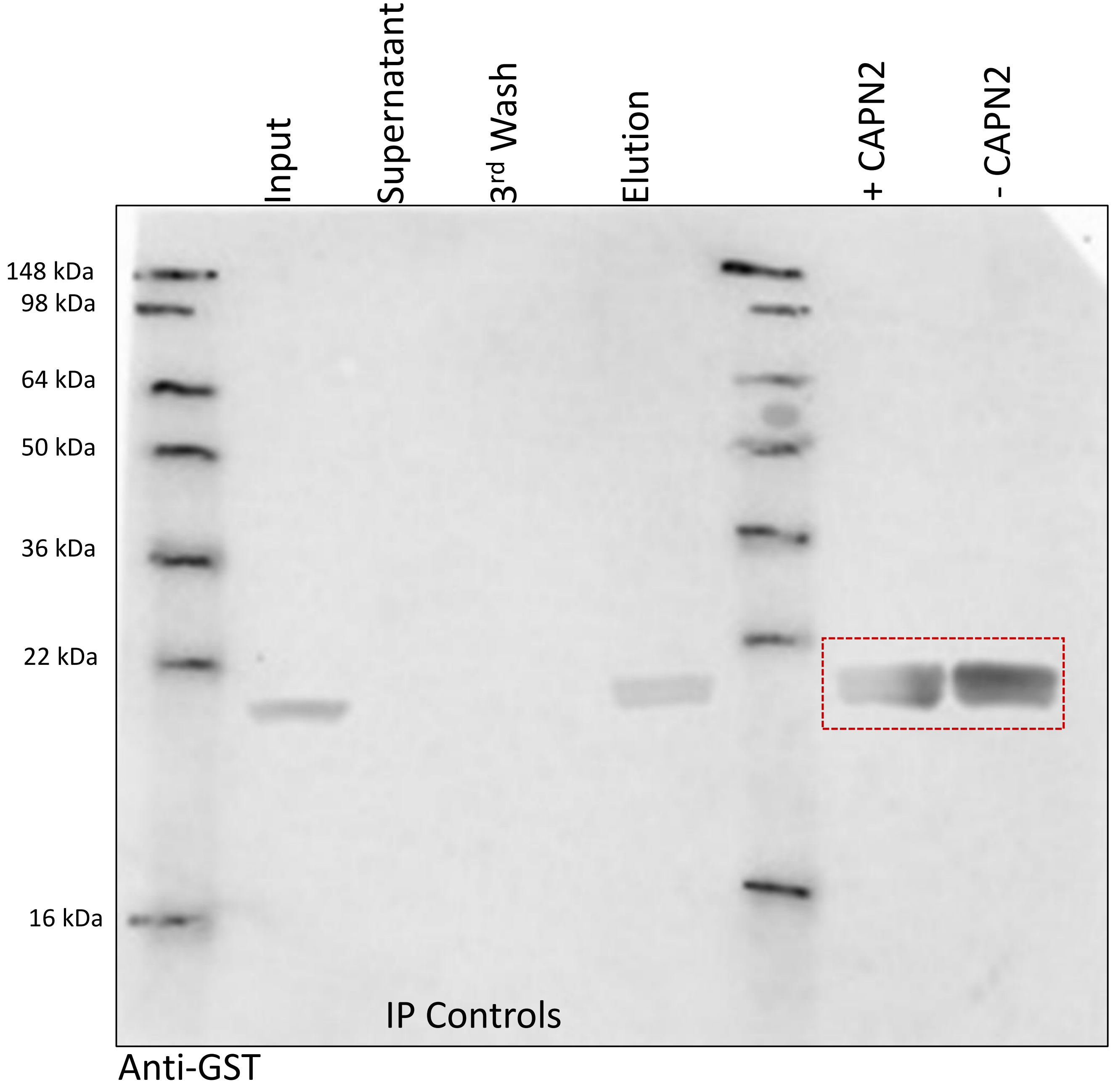

Journal of Cell Science • Supplementary information

Table S1. Antibodies for Western Blotting

Target	Company	Cat. No	Dilution
MEIS2	LifeSpan Biosciences	C31141	1:2.000
aTUB	abcam	ab4074	1:5.000
CAPN2	abcam	ab39165	1:2.000
HA-HRP	Sigma	12013819001	1:10.000
LaminB1	abcam	ab16048	1:1.000
PBX1b	Santa Cruz	sc101852	1:5.000
GFP	abcam	ab290	1:1.000
GST-HRP	Sigma	GERPN1236	1:20.000

Table S2. Primary and secondary antibodies for ICC

Target	Company	Cat. No	Dilution
Primary			
DCX	Merck	AB2253 / Sigma Aldrich	1:5.000
Flag	Sigma	F3156	1:1.000
GFP	abcam	ab290	1:2.500
TurboGFP	Invitrogen	PA5-22688	1:3.000
MEIS2 C-term.	Sigma	WH0004212M1	1:2.000
MEIS2 N-term (Alexa 488)	Santa Cruz Bio-technology	sc-515470	1:500
PBX1b	Santa Cruz Bio-technology	sc101852	1:50
Secondary			
goat anti-rabbit Alexa488	Invitrogen	A11008	1:1.000
goat anti-guinea pig Alexa568	abcam	ab175714	1:1.000
goat anti-mouse Alexa568	Invitrogen	A11004	1:1.000
goat anti-rabbit Alexa647	Invitrogen	A21244	1:1.000
goat anti-guinea pig Alexa647	Invitrogen	A21450	1:1.000
Counterstain			
DAPI	Invitrogen	D1306	1:10.000
Phalloidin iFluor647	abcam	ab176759	1:10.000

Journal of Cell Science • Supplementary information

Table S3. Primer pairs for calpain transcript profiling

Target mRNA	Orientation	Sequence
Beta-actin	for	AGCCATGTACGTAGCCATCC
	rev	CTCTCAGCTGTGGTGGTGAA
Capn1	for	GCCAGCAAGAAGCTGCAGCCCG
	rev	CCTTAAGAGCAGGACCCTCCGG
Capn1	for	GACGTGGTCATAGATGACTTGC
	rev	GTAAAGTCCTCGAAGGCCTCTG
Capn2	for	GCGAGGACATGCACACCATTGGC
	rev	GCTTGAAGCGGTTGAGGACCTCG
Capn2	for	GGTCCGTCTGGAAACGCTATTC
	rev	CTAGACCTCTCCAGGCATGTAAC
Capn3	for	GGGTGAGGGGCTGTTCTGCTG
	rev	CGCCGGTTCTTCTGCATCAGAGC
CAST	for	GAAGTGTGGTGAAGATGAGGAC
	rev	GGAGTTTGGGATGTGTCATTGG
Capn4/Capns1	for	GAGAGTGAAGAAGTCCGTCAG
	rev	CCATCAGTTTTCAGATCCGGG
Capns2	for	GTCGTGGAGGCCTCAGAAAG
	rev	GGTCAGCTGCAGCCATTCTC
Capn5	for	GCCCACAGTCAACAACCAGC
	rev	CAGCACTCGCTCAAAGAGCTG
Capn6	for	GAAGAAGGGCAGCTACGTGATTG
	rev	CATACTTCTTCTCCAGGCCCTC
Capn7	for	GATGCTTATAGCCTGGCCAAC
	rev	CCCATCAATGTATGGAGGTGG
Capn8	for	CTTCCTACACTCCGAAGAAGG

Journal of Cell Science • Supplementary information

	rev	CTGCTTCGGCTGCGTTTGAG
Capn9	for<br>rev	CTTACCTGCATCGGTCCCTG<br>GAGGGTTAGGGAGGCAATGG
Capn10	for<br>rev	TGTTTCCAGATAACCCATGGG<br>CCGACAAGTTCTGTGCTCCC
Capn11	for<br>rev	CAGTTCAAGATCTCTCTCCCG<br>CCCGGTATTTCAAGAAGAAGTC
Capn12	for<br>rev	GACGATGATGAAGAGGGACC<br>CTGGGTACCACCAGGTAGTG
Capn13	for<br>rev	CCTGATGGACCAAAGCTATTCG<br>GTAATTGGGAATAGGAGCCAG
Capn15	for<br>rev	GACACTGATCTCATCTGGGC<br>CAACTGCCATTCCAGGAGAAAC

Journal of Cell Science • Supplementary information

Table S4. Primer pairs for site directed mutagenesis

Template	Mutation	sequence	Orient.
mMeis2b	S^{198}-E^{198}	AGAGATGGAAGCTCCAAGGAAGATCATGAAGAACTTTCA<br>CTCATCAATCACGAGGTCAATGGGCATTTTTCCTGTGGA	for<br>rev
mMeis2b	S^{198}-E^{198} 4xPhos	AGAGATGGAAGCTCCAAGGAAGATCATGAAGAACTTTCA<br>GCGAGATTTGTGGAGGAGCCTTCCAGTTCTTC<br>GCGAGATTTTCGGAGTCGCCTTCCAGTTCTTC	for<br>rev1<br>rev2
mMeis2b	S^{261}-D^{261}	GTGATGACGACGATCCAGACAAGGAC<br>CTGTGCCAGGATCAGCTACGCTGTTG	for<br>rev
mMeis2b	T^{264}-D^{264}	CTGATCCTGGCGACGGTGATGACGAC<br>CTACGCTGTTGTCTAACCCATCGCC	for<br>rev
mMeis2b	S^{261}-A^{261}	GTGATGACGACGATCCAGACAAGGAC<br>CTGTGCCAGGTGCAGCTACGCTGTTG	for<br>rev
mMeis2b	T^{264}-I^{264}	CTGATCCTGGCATAGGTGATGACGAC<br>CTACGCTGTTGTCTAACCCATCGCC	for<br>rev
hCAPN2	S^{50}-D^{50}	CCTGGGGGACTCCTGGCTGCTGGCTG<br>GCTCCTTGGCAGATGTCTGTGCGGGTG	for<br>rev

Table S5. Plasmids

Name	Source
pGIPZ-shRNA Capn4	Dharmacon
pGIPZ-non silencing shRNAmir RH4346	Dharmacon
pCLIG-Pax6	Hau et al., Scientific Reports (2021) 11:21013
pCLIG-Pbx1	Hau et al., Scientific Reports (2021) 11:21013
pSF91-Capn2	This study
pSF91-Capn2^{S50D}	This study
pWPI-Meis2	This study
pWPI-Meis2^{4xPhos}	This study
pWPI-Meis2^{AI}	This study
pCLIG-Meis2^{AI}	This study
pCLIG-Meis2^{DD}	This study

Journal of Cell Science • Supplementary information

Table S6. Software

Name	Identified, Reference
GPS-CCD (group-based prediction system-calpain cleavage detector)	Liu et al., 2011; http://ccd.biocuckoo.org/); RID: SCR_000202
PESTfind	https://emboss.bioinformatics.nl/cgi-bin/emboss/epestfind
ImageJ/Fiji software	RRID: SCR_003070
BioVoxxel Toolbox	RRID: SCR_015825
GraphPad Prism version 7.0	RRID: SCR_002798
Clustal omega multiple sequence alignment	RRID:SCR_002909

Journal of Cell Science • Supplementary information

Table S7. ImageJ/BioVoxxel script for image analysis.

Location and file format	input = "C:/Users/Marin/Desktop/Marina/MasterThesis/Results/ImageJ_analysis_neurogenesis/input/"<br>output = "C:/Users/Marin/Desktop/Marina/MasterThesis/Results/ImageJ_analysis_neurogenesis/output/"<br>suffix = ".nd2"
Function to proceed as batch analysis through input folder scanning all files with correct suffix	processFolder(input);<br>function processFolder(input) {<br>list = getFileList(input);<br>list = Array.sort(list);<br>for (i = 0; i < list.length; i++) {<br>if(File.isDirectory(input + File.separator + list[i]))<br>processFolder(input + File.separator + list[i]);<br>if(endsWith(list[i], suffix))<br>processFile(input, output, list[i]);<br>}<br>}
Open files	function processFile(input, output, file) {<br>run("Bio-Formats Windowless Importer", "open=" + input + file + " autoscale color_mode=Default rois_import=[ROI manager] view=Hyperstack stack_order=XYCZT");
Rename original file in neutral way, duplicate GFP-channel and convert to 8-bit format	rename("nikon_image");<br>run("Duplicate...", "title=GFP duplicate channels=2");<br>setOption("ScaleConversions", true);<br>run("8-bit");<br>run("Duplicate...", "title=GFP_filter");
Apply Threshold Filter Algorithm on GFP-channel; Note: Threshold method is variable and was optimized for each condition and experiment (see Table)	run("Auto Threshold", "method=**Otsu** white");
Duplicate DAPI-channel and convert to 8-bit format	selectWindow("nikon_image");<br>run("Duplicate...", "title=DAPI duplicate channels=1");<br>setOption("ScaleConversions", true);<br>run("8-bit");<br>run("Duplicate...", "title=DAPI_filter");
Apply Threshold Filter Algorithm on DAPI-channel; Note: always Triangle (see Table)	run("Auto Threshold", "method=**Triangle** white");
Create intersection of GFP- and DAPI-channel	imageCalculator("AND create", "GFP_filter","DAPI_filter");<br>selectWindow("Result of GFP_filter");

Journal of Cell Science • Supplementary information

using Boolean operation ‘AND’, rename, duplicate	rename("intersection_DAPI+GFP");<br>run("Duplicate...", "title=intersection_smoothed");
Smoothing via ‘Erode’, ‘Dilate’ and ‘Watersheding’, duplicate	run("Options...", "iterations=2 count=1 black do=Erode");<br>run("Options...", "iterations=2 count=1 black do=Dilate");<br>run("Watershed Irregular Features", "erosion=15 convexity_threshold=0 separator_size=0-Infinity");<br>run("Duplicate...", "title=GFP");
Count number of GFP-positive cells (pixel size 50-500)	run("Analyze Particles...", "size=50-500 pixel show=[Count Masks] display clear summarize add");
Duplicate DCX-channel and convert to 8-bit format	selectWindow("nikon_image");<br>run("Duplicate...", "title=DCX duplicate channels=3");<br>setOption("ScaleConversions", true);<br>run("8-bit");<br>run("Duplicate...", "title=DCX_filter");
Apply Threshold Filter Algorithm on DCX-channel; Note: always Yen (see Table)	run("Auto Threshold", "method=**Yen** white");
Create intersection of DCX- and DAPI-channel using Boolean operation ‘AND’, rename, duplicate	imageCalculator("AND create", "DAPI_filter","DCX_filter");<br>selectWindow("Result of DAPI_filter");<br>rename("intersection_DAPI+DCX");<br>run("Duplicate...", "title=[intersection_DAPI+DCX smoothed]");
Smoothing via ‘Erode’, ‘Dilate’ and ‘Watersheding’, duplicate	run("Options...", "iterations=2 count=1 black do=Erode");<br>run("Options...", "iterations=2 count=1 black do=Dilate");<br>run("Watershed Irregular Features", "erosion=15 convexity_threshold=0 separator_size=0-Infinity");<br>run("Duplicate...", "title=DCX");
Count number of DCX-positive cells (pixel size 50-500)	run("Analyze Particles...", "size=50-500 pixel show=[Count Masks] display clear summarize add");
Create intersection of GFP/DAPI and DCX/DAPI intersections using Boolean operation ‘AND’, rename, duplicate	imageCalculator("AND create", "intersection_DAPI+GFP","intersection_DAPI+DCX");<br>selectWindow("Result of intersection_DAPI+GFP");<br>rename("intersection_DAPI+GFP+DCX");<br>run("Duplicate...", "title=[intersection_DAPI+GFP+DCX smoothed]");
Smoothing via ‘Erode’, ‘Dilate’ and ‘Watersheding’, duplicate	run("Options...", "iterations=2 count=1 black do=Erode");<br>run("Options...", "iterations=2 count=1 black do=Dilate");<br>run("Watershed Irregular Features", "erosion=15 convexity_threshold=0 separator_size=0-Infinity");<br>run("Duplicate...", "title=DCX+GFP");
Count number of GFP/DCX double positive cells (pixel size 50-500)	run("Analyze Particles...", "size=50-500 pixel show=[Count Masks] display clear summarize add");

Save summary window as csv-file	selectWindow("Summary");<br>saveAs("Results", output + file + ".csv");<br>run("Close");<br>run("Close All");<br>if (isOpen("Results")){<br>selectWindow("Results");<br>run("Close");<br>}<br>print("Processing: " + input + File.separator + file);<br>print("Saving to: " + output);<br>}

NT-shRNA vs shCAPN4 (Figure 6)

	NT-shRNA (#693)	shCAPN4 (#878)
Diff1	GFP: *Otsu*<br>DAPI: *Triangle*<br>DCX: *Yen*	GFP: *Moments*<br>DAPI: *Triangle*<br>DCX: *Yen*
Diff2	GFP: *Huang2*<br>DAPI: *Triangle*<br>DCX: *Yen*	GFP: *Li*<br>DAPI: *Triangle*<br>DCX: *Yen*
Diff3	GFP: *Huang2*<br>DAPI: *Triangle*<br>DCX: *Yen*	GFP: *Li*<br>DAPI: *Triangle*<br>DCX: *Yen*

GFP vs MEIS2-4xPhos (Figure 6)

	GFP	MEIS2-4xPhos
Diff9	GFP: *Li*<br>DAPI: *Triangle*<br>DCX: *Yen*	GFP: *Li*<br>DAPI: *Triangle*<br>DCX: *Yen*
Diff10	GFP: *Li*<br>DAPI: *Triangle*<br>DCX: *Yen*	GFP: *Otsu*<br>DAPI: *Triangle*<br>DCX: *Yen*
Diff11	GFP: *Otsu*<br>DAPI: *Triangle*<br>DCX: *Yen*	GFP: *Otsu*<br>DAPI: *Triangle*<br>DCX: *Yen*

Journal of Cell Science • Supplementary information
